# Lipid droplets as endogenous intracellular microlenses

**DOI:** 10.1038/s41377-021-00687-3

**Published:** 2021-12-06

**Authors:** Xixi Chen, Tianli Wu, Zhiyong Gong, Jinghui Guo, Xiaoshuai Liu, Yao Zhang, Yuchao Li, Pietro Ferraro, Baojun Li

**Affiliations:** 1 Institute of Nanophotonics, Jinan University, 511443 Guangzhou, China; 2 Department of Physiology, School of Medicine, Jinan University, 510632 Guangzhou, China; 3 CNR-ISASI, Institute of Applied Sciences and Intelligent Systems «E. Caianiello», Via Campi Flegrei 34, 80078 Pozzuoli, Naples Italy

**Keywords:** Biophotonics, Imaging and sensing

## Abstract

Using a single biological element as a photonic component with well-defined features has become a new intriguing paradigm in biophotonics. Here we show that endogenous lipid droplets in the mature adipose cells can behave as fully biocompatible microlenses to strengthen the ability of microscopic imaging as well as detecting intra- and extracellular signals. By the assistance of biolenses made of the lipid droplets, enhanced fluorescence imaging of cytoskeleton, lysosomes, and adenoviruses has been achieved. At the same time, we demonstrated that the required excitation power can be reduced by up to 73%. The lipidic microlenses are finely manipulated by optical tweezers in order to address targets and perform their real-time imaging inside the cells. An efficient detecting of fluorescence signal of cancer cells in extracellular fluid was accomplished due to the focusing effect of incident light by the lipid droplets. The lipid droplets acting as endogenous intracellular microlenses open the intriguing route for a multifunctional biocompatible optics tool for biosensing, endoscopic imaging, and single-cell diagnosis.

## Introduction

With the demand in real-time monitoring of endoplasmic variations and rapid detection of extracellular signals, a great number of approaches to bioimaging have been developed, including electronic, X-ray, optical, ultrasonic, magnetic, thermal, and mechanical methods, to obtain the significant information about the physiological and pathological processes of cells^1–3^. Among these approaches, optical microscope imaging techniques become preferable as they enable a straightforward and real-time visualization that is highly desirable for observation and diagnostics in vivo. To improve the discernibility to cells and tissues in different species, fluorescence signals are usually employed in optical microscope imaging techniques, which provides the possibility of monitoring and detection of specific organelles and cells^4^. However, the fluorescence signals emitted from subcellular structures are generally very weak so that it becomes difficult to directly apply the fluorescent methods to intracellular imaging or detection for single-cell studies. Although the fluorescence intensity can be increased by increasing the power of excitation light, the risk of phototoxicity to cells becomes higher and the photobleaching effect becomes stronger at the same time^5–7^.

The past few decades have witnessed a dramatic progress in optical imaging^8–10^, especially with the emerging of microsphere-assisted techniques that enable real-time and super-resolution imaging at visible wavelengths with conventional optical microscope^8,11–16^. The microspheres also provide the opportunities to take advantage of some photonic functions such as directional antennas, whispering-gallery modes, and photonic nanojets to enhance the optical signals including fluorescence^13,17^, upconversion emission^18^, Raman scattering^13,19^, and backscattering^17,20^. However, most of the microspheres in current strategies are in solid and artificially synthetic materials (e.g., SiO_2_, polystyrene, BaTiO_3_, TiO_2_) and so they are of very low biocompatibility, which becomes a fundamental aspect of biomedical applications when contact with the sample is needed, for example in single-cell analysis in vivo or endoscopy^21^. Fortunately, lessons from nature have shown that some bio-components and already-existing objects (e.g., red blood cells, yeast cells, spheroid cells, spider silks) can interact with light and are able to take the functions as real optical elements (i.e. microlenses, resonators, waveguides, gratings or tools for photolithography)^22–31^. The lensing effect of some biological samples indicates the possibility to construct fully biocompatible microlenses with proper focal length and magnification to achieve real-time monitoring and detection of optical signals of a single cell^23–25^.

In this work, we demonstrate that lipid droplets, a sort of dynamic structures that naturally exist in cells^32^, can act as intracellular microlenses for real-time monitoring of subcellular structures and detection of extracellular signals. With a spherical shape and a refractive index higher than cytoplasm and periplasm^33,34^, the lipid droplets exhibit the lensing effect to efficiently converge both the excitation light and the fluorescence signals. Driven through optical tweezers, we show that single lipid droplets can be moved inside the cell to perform an enhanced intracellular fluorescence imaging of cytoskeleton. Moreover, we demonstrate that due to the refractive index contrast of the lipid droplets in respect to cytoplasm, the excitation light is suitably focused for enhancing the mitochondrial fluorescence signals from cancer cells in the extracellular fluid. Thus, we proof that the lipidic microlenses are very useful and real-world tools able to assist fluorescent imaging.

## Results

### Materials characterization, experimental setup, and lensing effect analysis

**Fig. 1:**
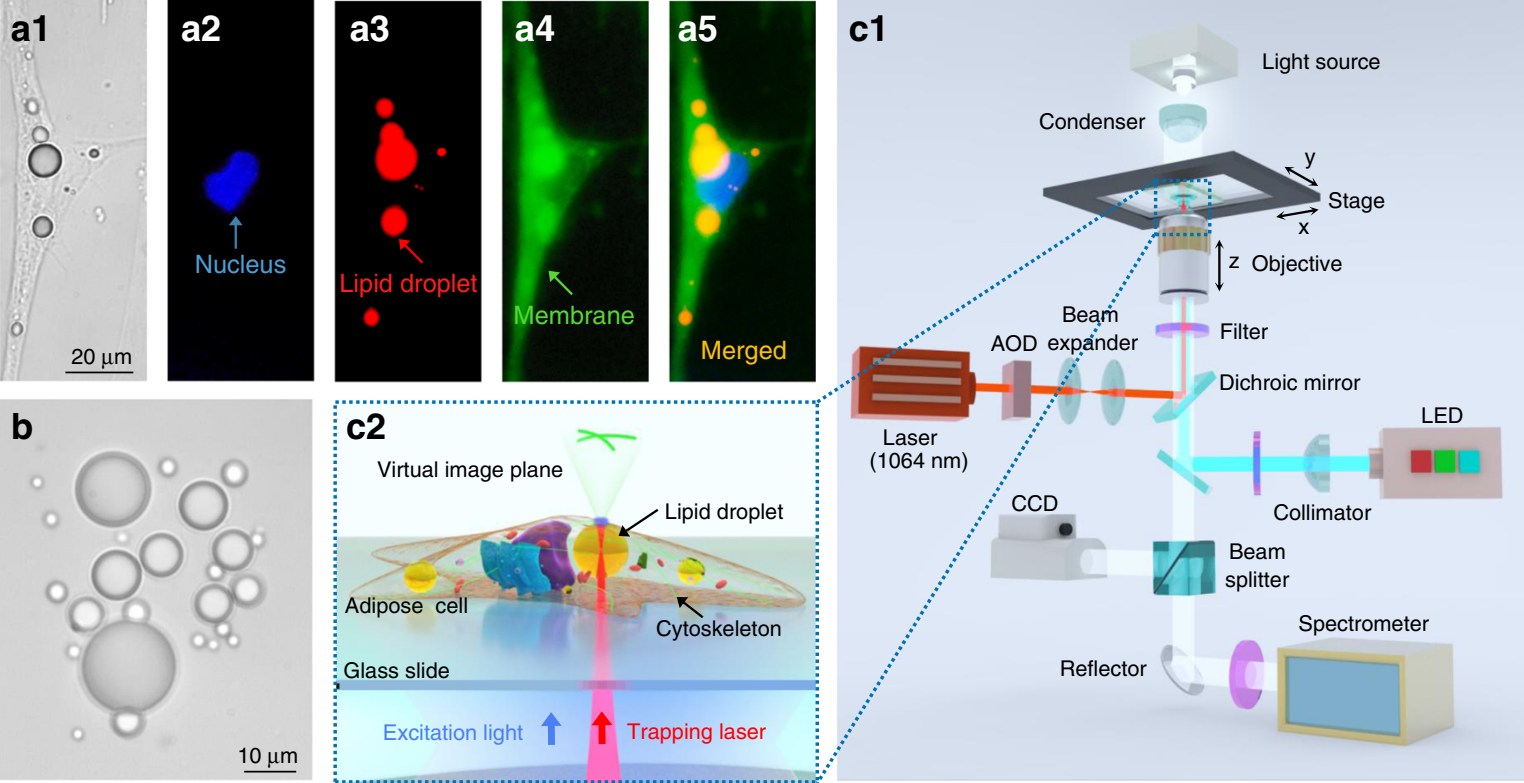
Materials and schematics of the intracellular microlenses. **a** Bright-field and fluorescence images of a fixed adipose cell. **a1** Bright-field image. **a2** Nucleus indicated by Hoechst 33342 (blue). **a3** Lipid droplets indicated by Oil-red O (red). **a4** Membrane indicated by DiO (green). **a5** Merged image of **a2**–**a4**. **b** Optical microscope image of the extracted lipid droplets in different sizes in vitro. (**c**) Schematics of the experiments. **c1** Experimental setup. **c2** An optically trapped lipid droplet inside an adipose cell focuses the excitation light and collects the fluorescent signals from the cytoskeleton to form a magnified fluorescence image

The lipid droplets used in this work were those naturally existing in commercially available human adipocytes-visceral cells (Fig. 1a, see “Methods” for cell culture). The droplet generally maintains a smooth surface, micrometer size (0.5–40 μm), and a spherical shape^35^ (Fig. 1b; also see Fig. S1 and Video S1 of the Supplementary Information). Meanwhile, the droplets exhibit a high transparency at visible wavelengths (see the transmittance spectrum in Fig. S2 of the Supplementary Information) and a refractive index of ~1.52 (see “Section” S1.1 of the Supplementary Information), higher than that of the cytoplasm (~1.36). With these morphology and optical properties, the lipid droplets are expected to act as a sort of endogenous lensing structures. The experimental setup was built around a scanning optical tweezing system (OTS) incorporated with an inverted optical microscope for detecting the bright-field and fluorescence signals (Fig. 1c1) (see “Methods” for the details of the experimental setup). The trapping light in the OTS was a laser beam (Gaussian beam) at the wavelength *λ* of 1064 nm that is within the biological window^36^. The range of a single optical trap created by the trapping light of the OTS, i.e., the spot size of the beam, is ~1.3 μm, and a single lipid droplet can be attracted and moved by a single optical trap at a time. With the assistance of such an OTS, the lipid droplets can be trapped and moved inside the cells, acting as transportable intracellular microlenses (Fig. 1c2).

**Fig. 2:**
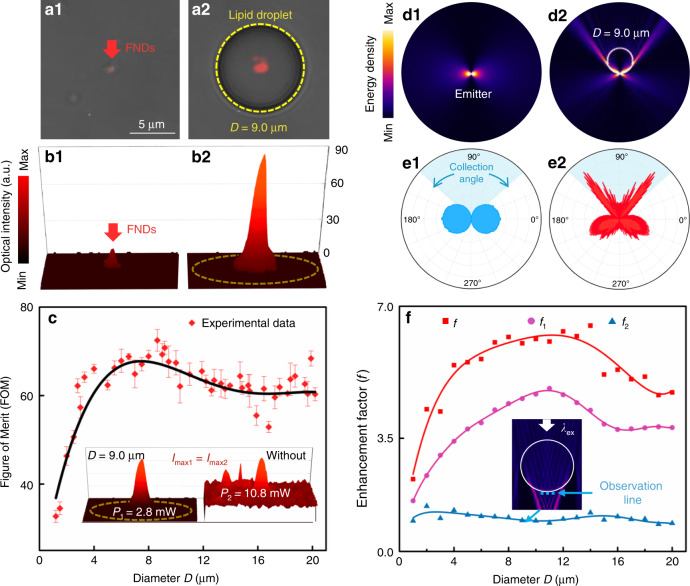
Lensing effect of the lipid droplets. **a** Images merged by bright-field and fluorescence images of the FNDs. **a1** Bare FNDs on a glass slide. **a2** FNDs with a lipid droplet (diameter: 9 μm) moved right below them. **b** Optical intensity distributions. **c** FOM as a function of the droplet diameter. The inset shows the optical intensity distributions of the FNDs with and without the 9-μm lipid droplet at different excitation powers (*P*_1_ and *P*_2_) with the same detected maximum fluorescent intensity. **d** Energy density distributions of the emitters without (**d1**) and with (**d2**) the lipid droplet. **e** The corresponding emission patterns. **f** Enhancement factor (*f*) as a function of the diameter of the lipid droplets. The inset shows the position of the observation line for the calculation of *f*_2_

In the case of intracellular imaging, the objects were generally underneath lipid droplets due to the intrinsic pressure inside the cell^37^ and so the droplets worked in a contact mode of microlenses^8^. To analyze their lensing effect in contact mode, the lipid droplets were extracted from the adipose cells to index matching liquid that was matched with the cytoplasm (~1.36). Bright-field imaging was performed on a grating sample (width: 650 nm; spacing: 350 nm), and a magnification of ∼2.1 can be obtained by using the ∼3.2 μm lipid droplet (see Fig. S3 of the Supplementary Information). A finer grating structure of a Blu-ray Disk (width: 200 nm, spacing: 100 nm) was also used as a sample for imaging in bright field (see Fig. S4a1, b of the Supplementary Information). Without the lipid droplet in the field of view of the optical microscope (objective ×60, NA = 1), the gratings of the disk could not be resolved. With the presence of the lipid droplets, the adjacent gratings became discernible. The results indicate that in contact mode, a feature size of 100 nm can be resolved with the assistance of the lipid droplets. A possible mechanism of the gain in imaging ability is that the near-field evanescent waves that carry high spatial frequency information of the sample are coupled into the lipid droplets and transformed into propagating waves for microscopic observation^38,39^. Besides, the presence of the lipidic microlenses reduces the full width at half maximum of the point spread function of the imaging system^40^, which also contributes to the improved imaging performance of the system. By taking the advantages of the lensing effect in contact mode, the bright-field imaging and fluorescence detection of bacteria (see Methods for cell culture and preparation) and even virus (see “Methods” for fluorescence labeling and infection) have also been sufficiently improved (see Fig. S4a2, c, d of the Supplementary Information). In particular, to investigate the fluorescence enhancement by the lipid droplets, fluorescent nanodiamonds (FNDs) prepared with a solvent evaporation method (see Section S1.2 of the Supplementary Information) were used as fluorescence emitters to be enhanced. Attributed to the photobleaching-resistance, photostability^41^, and the linear relationship between the emission intensity and the excitation power (see Fig. S5 of the Supplementary Information), the fluorescence enhancement experiments were repeated with the same FNDs for the lipid droplets of different diameters. The FNDs placed at the top of the index matching liquid with freely suspended lipid droplets were excited by the light through a bandpass filter (540–580 nm) from the light-emitting diode (LED). The excitation light, with a power set at ~2.8 mW (intensity: 22 mW mm^−2^), was converged by the inverted objective below the chamber. As shown by the merged images (bright-field and fluorescent) in Fig. 2a, without the lipid droplets (Fig. 2a1), a very weak signal of red fluorescence was detected from the position of the FNDs (Fig. 2b1). By contrast, with the assistance of a lipid droplet (i.e. with a diameter of 9.0 μm) that was moved right below the FNDs (Fig. 2a2), a sufficiently enhanced fluorescent signal (~6 folds) was detected (Fig. 2b2). Note that for the consistency with the case of the fluorescent emitters and the microlenses in living cells (to be presented in later sections), the lipid droplet was set almost in contact with the FNDs. The enhancement by the lipid droplets indicates that the optical power required for the excitation light can be efficiently reduced to achieve the fluorescent imaging with the assistance of the lipid droplets. To investigate the capacity of the lipid droplets to reduce the excitation power, the droplets of different diameters *D* (from 1 to 20 μm) were used to enhance the intensity of detected fluorescence and to obtain the fluorescent images of the FNDs (Fig. 2c). In each of the measurements and imaging processes, an optical power *P*_1_ was applied for exciting the targeted FNDs with the assistance of the lipid droplets and the maximum of the detected fluorescence intensity was *I*. Then a power *P*_2_ was applied to excite the same FNDs without the lipid droplets was adjusted to obtain a maximum of the fluorescence intensity that was equal to *I*. A figure of merit (FOM) is thereby defined as FOM = (*P*_2_ – *P*_1_)/*P*_2_ × 100%. As shown in Fig. 2c, the FOM values were larger than 35% for the droplets of 1–20 μm, and a maximum of the FOM (73%) can be obtained at *D* = 9 μm. The results indicate that the presence of the lipid droplets significantly reduces the needed excitation power, thus decreasing the possible photobleaching and photo-damage to the sample.

In order to preliminarily evaluate the fluorescence enhancement by the lipid droplets in the contact mode, numerical simulations by finite element method of the field distributions were performed (see “Methods” for the simulation details). The FNDs were considered as point sources (emitters) that have both longitudinally and transversely polarized components. In the longitudinal polarization, the direction of the dipole was perpendicular to that of the objective, resulting in a very low collection efficiency by the objective (~10.7%). Therefore, the longitudinally polarized dipole is discussed here as an extreme circumstance (Fig. 2d1). Note that in the transversal polarization, as the direction of dipole was parallel to the direction of the objective, most of the light has been collected directly by the objective and the improvement in collection efficiency by the lipid droplets is much weaker than that in the longitudinally polarized dipole (see section S1.3 and Fig. S6 of the Supplementary Information). A lipid droplet of 9 μm was above and in contact with the emitter to act as a microlens (Fig. 2d2). The emission patterns for the emitters without and with the lipid droplet were then obtained by the simulated field distributions in the far field (50 μm) above the emitter, as shown in Figs. 2e1 and 2e2, respectively. The blue shades in Fig. 2e represent the collection angle (±47.3°) of the microscope objective used in the experiments. It can be seen that with the lipid droplet, the emission becomes highly directional (with a divergence angle decreased from 180° to about 110°) so that the emission is redirected to the objective. The collection efficiencies, defined as the ratio of the energy collected by objective to the total energy of the emission, were calculated to be 10.7% and 48.5% for the emitters without and with the lipid droplet (*D* = 9 μm), respectively. The enhancement factor *f*_1_ for the collection efficiency, defined as the ratio of the collection efficiencies with the droplets to those without the droplets (*D* from 1 to 20 μm), was estimated and presented by the purple dots in Fig. 2f. Since the fluorescence enhancement was also attributed to the focusing of the excitation light, the enhancement factor *f*_2_ for the excitation light is defined as *f*_2_ = *I*_2_/*I*_1_, where *I*_1_ and *I*_2_ are the average intensities at the observation lines (indicted by the blue dashed line in the inset of Fig. 2f), for the cases without and with the lipid droplets, respectively. In contact mode, the observation line was set in the near-field of lipid droplets. However, as the focusing region of the excitation light was beyond the near-field range of the lipid droplets, the values of *f*_2_, as presented by the blue dots in Fig. 2f, were much smaller than those of *f*_1_. The total enhancement factor *f* of the lipidic microlenses can then be calculated by *f* = *f*_1_ × *f*_2_^42^, and the results are presented by the red dots in Fig. 2f. It is indicated that the major contribution to the fluorescence enhancement was the increased collection efficiency by the lipid droplets acting as microlenses in the contact mode. Therefore, the proposed strategy exploits improving the signal collection instead of increasing the excitation power, reducing the risk of photobleaching and photo-damage to cells in live-cell imaging. Furthermore, the lipid droplets can be extracted and patterned to form 2D microlens arrays and moved rapidly to perform a scan with the OTS followed by a reconstruction of the image by stitching the collected signals (see Fig. S7 of the Supplementary Information), which is a feasible method to overcome the small field of view and the intrinsic spherical aberrations of microlenses and to achieve high-throughput in scanning imaging and signal detecting in vitro.

### Intracellular imaging of subcellular structures

**Fig. 3:**
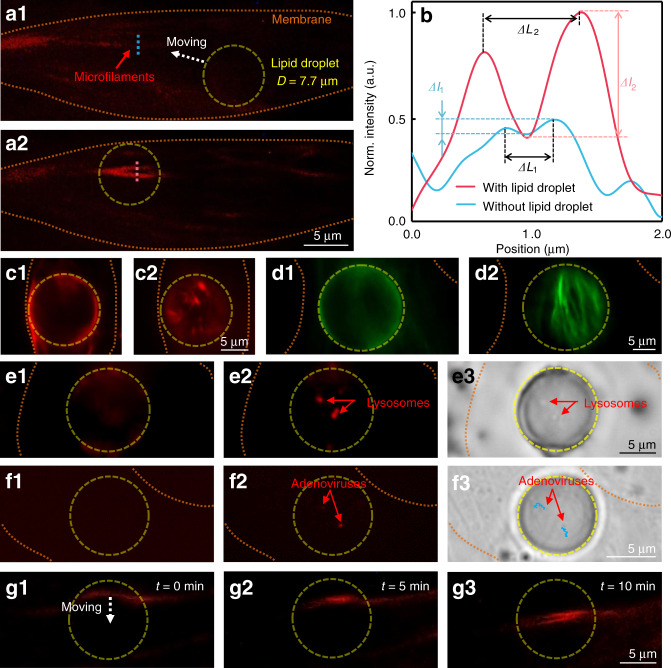
Intracellular imaging of subcellular structures. **a** Fluorescence images of the microfilaments (F-actin filaments) of a living adipose cell. The weak fluorescent signals (**a1**) were efficiently enhanced by the lipid droplet (diameter: 7.7 μm) (**a2**). **b** Normalized intensity distribution along the observation lines through microfilaments without (blue dashed in **a1**) and with (red dashed in **a2**) the lipid droplet. **c** Fluorescence images of the microfilaments in a living adipose cell with a 12.5-μm lipid droplet. **d** Fluorescence images of the microfilaments in a fixed adipose cell with an 18.6-μm lipid droplet. **e** Fluorescence and bright-field images of the lysosomes in a living adipose cell with an 11.3-μm lipid droplet. **f** Fluorescence and bright-field images of the adenoviruses in a living adipose cell with an 8.1-μm lipid droplet, the blue lines represent the movement trajectory of the adenoviruses in 10 min. The fluorescence images were focused on the surfaces of the cells (**c1**, **d1**, **e1**, and **f1**) and the virtual image planes (**c2**, **d2**, **e2**, and **f2**) formed by the lipid droplets. The bright-field image was focused on the virtual image plane formed by the lipid droplet (**e3**, **f3**). **g** The microfilament movement monitored by the lipid droplet (diameter: 10.0 μm) inside the cell in 10 min

By using the endogenous lipid droplets, intracellular imaging was then performed on several subcellular structures in living mature adipose cells. Within the life cycle of the cell, the endogenous lipid droplets vary in terms of their number, size, spatial organization, and the intracellular positioning^35^. With the optical traps created by the OTS, the position in which the lipid droplet can be deployed is confined within a certain region into the live cells. The trapped lipid droplet thus can act as a movable intracellular microlens, allowing us to achieve real-time imaging and detect the fluorescent signals of the field of interest (e.g. the subcellular structures in Fig. 1c2). With its microfilaments (F-actin filaments) labeled by SiR-actin (red, emission wavelength: 674 nm), a fluorescent living mature adipose cell containing a lipid droplet (*D* = 7.7 μm) was excited by green light (540–580 nm) at an optical power of 2 mW (intensity: 16 mW mm^−2^) (Fig. 3a1). Without the assistance of the lipidic microlens, the targeted microfilaments cannot be resolved through the microscope imaging system even in the optimal focal plane. When the lipidic microlens was trapped and then moved toward the targeted microfilaments by the OTS, the fluorescent signals were efficiently enhanced and the fluorescent image of the branch-shaped microfilaments was clearly visible (Fig. 3a2). Normalized intensity distribution was obtained along the observation lines through microfilaments without (blue dashed in Fig. 3a1) and with (red dashed in Fig. 3a2) the lipid droplet (Fig. 3b), indicating that the resolving ability of the imaging system was greatly improved by the lipid droplet. With the peaks of the intensity distributions through the microfilaments, the magnification (*M*) was calculated as *M* = Δ*L*_2_/Δ*L*_1_ ≈ 2, where Δ*L*_1_ and Δ*L*_2_ are the separation between the peaks of the systems without and with the assistance of the lipid droplet, respectively. Also, the contrast *C* of the fluorescent signals has been increased by the lipid droplet. The contrast *C* can be expressed as *C* = Δ*I*/*I*_max_, where *I*_max_ is the maximum fluorescent intensity of the peaks and Δ*I* is the drop from the peaks to the valley between them. Without the lipid droplet, the calculated contrast was 14.8%, below the Rayleigh criterion of 26.4%^43^. Instead, with the lipidic microlens, the contrast was increased to 62.5%.

Further experiments show that the lipid droplets can be used to improve the fluorescent imaging of microfilaments in both living and fixed cells (Fig. 3c, d), and a larger field of view can be achieved with the lipid droplets in larger sizes. Besides the microfilaments, the enhancement in the imaging by the lipid droplets is also exploitable for the lysosomes in living cells in both fluorescence (Fig. 3e1, e2) and the bright-field (Fig. 3e3) modes. The lysosomes were labeled by Lyso-Tracker (red) while the excitation power was 1.5 mW (intensity: 12 mW mm^−2^). Furthermore, with the feature of long-term existence (steady state) inside the mature adipose cells^35^, the lipid droplets can be used to monitor the dynamic process and physiological state of the cell. The movement of the adenoviruses was monitored by the lipid droplet (*D* = 8.1 μm) inside the cell at an excitation power of 5 mW (intensity: 40 mW mm^−2^) (Fig. 3f), and the movement trajectories of the infection process of adenoviruses were recorded in 10 min (Fig. 3f3). The microfilament movement was also monitored by the lipid droplet (*D* = 10.0 μm) inside the cell at an excitation power of 2 mW (intensity: 16 mW mm^−2^) (Fig. 3g). The targeted microfilaments gradually moved from the edge to the center of the field of view covered by the lipid droplet in 10 min. Note that the fluorescence image of cells obtained by a conventional optical microscope is the 3D signals superimposed upon a 2D image. The overlap of signals on the z direction leads to the difficulty in resolving the details of subcellular structures and consequently a low signal-to-noise ratio. As the lipid droplets naturally existing inside the cells are very close to the subcellular structures, most of the fluorescent signals from the subcellular structures can be collected by the lipid droplets, which allows us to focus on the physiological processes of the subcellular structures (Fig. 3a, c–e, g) or even virus (Fig. 3f) on a specific plane and suppress the noise signals from non-target regions.

### Non-contact mode and detecting extracellular signals

**Fig. 4:**
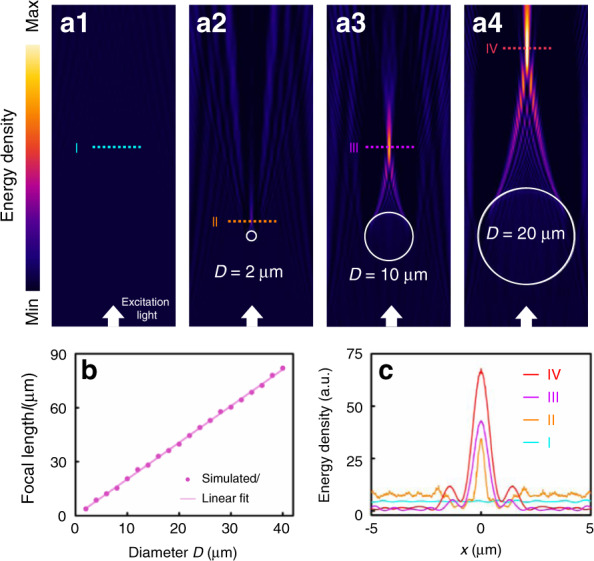
Concentration of excitation light in non-contact mode. **a** Energy density distributions for an incident plane wave as the excitation light (wavelength: in 475 nm) without the lipid droplet (**a1**) and with the lipid droplets of 2 (**a2**), 10 (**a3**), and 20 μm (**a4**) diameters. **b** The focal length *l* as a function of the diameter *D* at the wavelength of 475 nm. **c** Energy density distributions along the observation lines indicated in (**a**)

In addition to the contact mode in which the collection efficiency is the major contribution to the fluorescence enhancement, the long focal length of the lipid droplets can extend the fluorescence enhancement strategies to non-contact mode, thereby increasing the working distance of imaging and detection of lipid droplets. In the non-contact mode, the concentration of excitation light becomes the leading contribution so that the detection range of lipid droplets can be extended to the extracellular microenvironment and surrounding tissues. As indicated by the simulated field intensity distributions shown in Fig. 4a, with a plane wave as the excitation light (*λ*: 473 nm), the focal length *l* becomes larger with the increase of the lipid droplet diameter. For the diameters larger than 2 μm, the excitation light is focused at *l* > 10*λ* (Fig. 4b), and the intensity at the focus is also increased by the lipid droplet (Fig. 4c), suggesting a non-contact mode of such bio-microlenses in fluorescence. In this mode, the enhancement factor *f* of the lipid droplet is calculated given that target of detection is at the focus of the microlens. It is found that the enhancement factor related to the excitation intensity increases with droplet diameter, but the factor related to the collection efficiency slightly contributes to the total enhancement factor as the near-field collection of the lipid droplet becomes ineffective due to the long distance between the lipid droplet and the emitter (see Fig. S8 of the Supplementary Information). The lensing effect over a long distance in the non-contact mode provides an opportunity to enhance the fluorescence from the extracellular environment surrounding the cells. As reported^44^, in addition to brain vessels, periadventitial adipose cells are distributed ubiquitously around the blood vessels throughout the body, especially around arteries. Therefore, we used the lipid droplet in adipose cells to monitor the blood vessels around the cells. In the experiments, a glass capillary (wall thickness: 4 μm; inner diameter: 45 μm) was employed to act as the blood vessel that was adhered to the surface of an adipose cell (see Fig. S9 of the Supplementary Information). The objective of the microscope was adjusted to be aligned with the centers of both the capillary and the lipid droplet (*D* = 20.0 μm) in the living cell. As the focal length of the lipid droplet (*D* = 20.0 μm) is ~40 μm according to the simulations, the detection range of the lipid droplet can cover the capillary in the radial direction, and the focusing effect of the lipid droplet is not affected by the presence of the capillary (see Fig. S10 of the Supplementary Information).

**Fig. 5:**
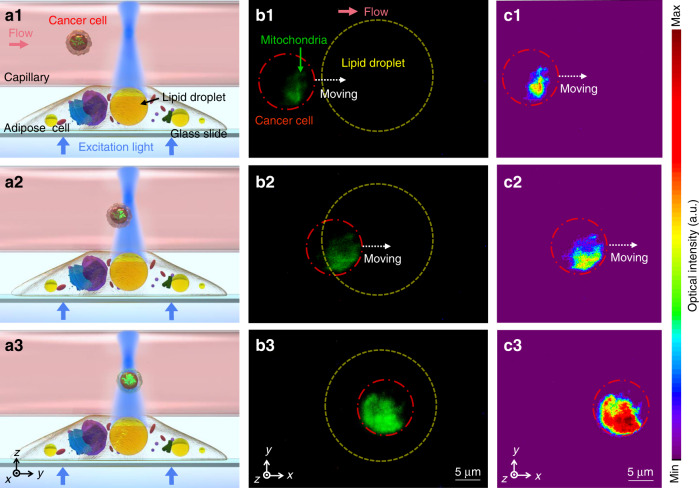
Detecting extracellular signals. **a** Schematic (side view) of the enhanced detection of cancer cells in fluid by a lipid droplet inside the adipose cell. **b** Fluorescence images (top view) of the detecting process. The yellow and red dotted circles indicate the lipid droplet (diameter: 20 μm) and the cancer cell, respectively. The mitochondria of the cancer cell were marked by Mito-Tracker (green). The red arrow indicates the direction of fluid flow in the capillary. **c** The corresponding intensity mapping of the fluorescence emitted from the cancer cell mitochondria

The excitation light was transmitted to the cell by the inverted objective and converged through the lipid droplet into the capillary (Fig. 5a1). The solution that contained blood cells and cancer cells (K562) flowed into the capillary. The mitochondria in the cancer cells were fluorescently labeled by Mito-Tracker (green) dyes as the targets of detection. The cancer cell flowing with the rightward fluid was gradually excited when it flowed through excitation light converged by the lipid droplet (Fig. 5a2, a3). Before reaching the effective region of the droplet, the fluorescence from the cancer cell was very weak (Fig. 5b1). As it arrived at the edge of the effective region, the fluorescence was enhanced a bit by the collection efficiency of droplet (Fig. 5b2). When the cancer cell reached the center of the effective region, the excitation light was highly concentrated, allowing us to obtain a much stronger fluorescent signal (Fig. 5b3, also see Video S2 of the Supplementary Information). As presented by the intensity mapping of the fluorescence from the cancer cell mitochondria (Fig. 5c), the signal strength of the fluorescence was greatly enhanced to improve the responsivity of the detection of cancer cells.

## Discussion

As organelles, endogenous lipid droplets can persist in the cytosol for a time that is dependent with the physiological state and the type of the cells. Generally, the mature lipid droplets with a larger size have a more stable morphology, which benefits the function as intracellular microlenses. However, due to the crowded intracellular environment, a higher optical power is required for the trapping laser of the OTS to manipulate the larger lipid droplets inside the cells (see Fig. S11 of the Supplementary Information). In our experiments, we can move the lipid droplets with a diameter of less than 8 μm inside the mature adipose cells. In the solution, the freely suspended lipid droplets trapped by the OTS can move in synchrony with the motion of the trap, as long as the trapping force on the lipid droplet was not exceeded by a drag force from the flow (see Section S1.4 and Fig. S12 of the Supplementary Information). Due to the much stronger drag force caused by the crowded intracellular environment, the movement of the trapped lipid droplets to the target position inside the cells becomes much slower (0.001–5 μm s^−1^). Note that an arbitrary shift of intracellular structures may affect the functions of the organelles and the interactions between subcellular components. To investigate the effect on cells by the moving of the lipid droplets, an experiment was carried out to indicate the cell viability after trapping and moving of the lipid droplets in adipose cells (see Fig. S13 of the Supplementary Information). In each cell, more than 3 lipid droplets were moved over an average distance of 5 μm. Untreated cells were included as a control group. The cells were then continuously cultured for 24 h and the survival rates were recorded every 8 h. In addition, the cell viability after cultivation for 8, 16, and 24 h was judged by staining the cells with the cell permeable nucleus counterstain (Hoechst 33342). The results show that the survival rates of treated cells were very close to those of the control group. Besides, no apparent morphological changes were observed after moving the lipid droplets, and the treated cells kept growing and storing lipids after 24 h.

The lipid droplets can also be applied to different species of cells by endocytosis (see Fig. S14a–d of the Supplementary Information). The free lipid droplets were co-cultured separately with the macrophage, tumor, epithelial and myocardial cells, and then internalized by the cells through phagocytosis and pinocytosis. The transfer efficiency in the four types of cells for 24 h was obtained (see Fig. S14e of the Supplementary Information). The internalization of the lipid droplets did not impair the cell proliferation (see Fig. S14f of the Supplementary Information). There have been some methods to induce the formation and accumulation of lipid droplets in other cell types (non-adipocytes), such as myocardial cells^45^, monocytes^46^, astrocytes^47^, and epithelial cells^48^. The accumulation of lipid droplets in these non-adipocytes was mainly induced in biochemical ways. Actually, lipid overload occurs in nearly all types of cells when the cells are in the abnormal metabolism. Apart from adipocytes, hepatic stellate cells and the cone photoreceptors of some species are also rich in long-term storage spherical lipid droplets with diameters larger than 1 μm that can be used as microlenses. It is also worth noting that there are some other bio-components that can work similarly to the lipid droplets. For example, chloroplasts and red blood cells (RBCs) can act as microlenses and also enable the amplification in imaging as well as the enhancement in fluorescent signal detection. These microlenses in biomaterials can be applied to some scenarios different with those of the lipid droplets. Chloroplasts, as a sort of botanic organelles, can be employed inside the plant cells, while RBCs in the blood vessels of animals may act as adaptive optofluidic microlenses due to their intrinsic elastic properties^25^. Compared with chloroplasts and RBCs, the lipid droplets exhibit higher robustness and stability so that they can act as micolenses in different biological environments and can be internalized into cells of different types. Besides, with a higher refractive index and a nearly spherical shape, the lipid droplets provide a better performance in magnification and fluorescence enhancement (see Section S1.5 and Fig. S15 of the Supplementary Information).

The lensing effect of the lipid droplets is also valid in bulky biological tissues. We explored the application of the lipid droplets to porcine adipose tissue, and the surface morphology (bright-field) of the tissue was magnified by the lipid droplets (see Fig. S16 of the Supplementary Information), which implies the potential of the lipid droplets for in vivo operations. Moreover, there are some methods that may further improve the performance of lipid droplets by increasing the utilization efficiency of incident light. For example, some carotenoid pigments for refining the spectral sensitivity and improving color vision naturally exist in the cone lipid droplets within the photoreceptors of vertebrates^28,49^. The refractive index of these lipid droplets can be increased up to 1.6 by binding lipid-soluble pigments (see Fig. S17 of the Supplementary Information), which can be utilized to increase the index contrast and to improve the imaging ability. In addition, we found that some lipid droplets in nearly spherical shapes could also act as microcavities to support resonances in whispering-gallery modes (WGMs). With an optical fiber probe next to a spherical stained lipid droplet inside an adipose cell, 532-nm laser light was coupled into the lipid microcavity to generate the WGM effects (see Fig. S18 of the Supplementary Information). Numerical investigations show that when WGM excitation occurs, the high-order scattering mode can form comparatively narrow pattern in the virtual image plane, and the transformation of the evanescent waves to propagating waves can be enhanced by the WGM, both of which are crucial effects in super-resolution imaging^50^. Therefore, generating the WGM effects by the lipid droplets is also a possible approach to the further enhancement of the imaging resolution by microspheres.

To summarize, we employed endogenous lipid droplets in mature adipose cells to act as the intracellular bio-microlenses for fluorescence imaging and signal enhancement. In the contact mode, the collection efficiency of the emitted fluorescence signals was improved, which sufficiently reduced the optical power required for excitation light in fluorescence imaging of subcellular structures within living cells. In the non-contact mode, the excitation light was highly converged by the lipid droplets to enhance the fluorescence from the extracellular environment surrounding the cells in real time. The presented lensing effect of the lipid droplets is expected to find applications to fully biocompatible miniaturized tools for biosensing, endoscopic analysis, and single-cell diagnosis for endoscopy. The use of the lipid droplets as intracellular microlenses also provides opportunities to construct diverse endogenous photonic devices.

## Materials and methods

### Cell culture and staining

Human preadipocytes-visceral (HPA-v) cells (ScienCell, USA) were seeded at a density of 1 × 10^4^ cells cm^−2^ on glass-bottom Petri dishes (35 mm, Biosharp, Anhui, China) and allowed to proliferate in preadipocyte medium for 36–48 h at 37 °C and 5% CO_2_. The fully confluent cells were then induced to differentiate. The preadipocyte medium was replaced by the preadipocyte differentiation medium and the replacement was conducted every 2 days. The differentiation to mature adipose cells was completed after 6–12 days (see Fig. S19 of the Supplementary Information), and mature adipose cells can be maintained in adipocyte medium up to 7 days. The K562 cells (Procell, Hubei, China) were seeded at a density of 1 × 10^4^ cells cm^−2^ in T25 flask and allowed to proliferate in the complete medium (IMDM + 1% penicillin-streptomycin + 10% fetal bovine serum) for 30–48 h at 37 °C and 5% CO_2_. The K562 cells were passaged at constant density (1:2-1:4) every 2 days. The *Escherichia coli* (*E. coli*) expressing green fluorescent protein were grown in Luria-Bertani medium at 37 °C for 12 h. The *E. coli* were harvested and washed gently in phosphate-buffered saline solution (PBS). The *E. coli* were fixed by mixing 300 μl of cells with 700 μl of a 4% (wt–vol^−1^) solution of paraformaldehyde in PBS and the mixture was incubated on ice for 5 min. The *E. coli* were then washed twice in PBS for removing the fixative. Samples of 30 μl were placed on a poly-L-lysine-coated slide and air dried for 2 h.

To stain the lipid droplets and the F-actin filaments, the mature adipose cells were washed twice with DPBS (Dulbecco’s phosphate buffer saline) and then fixed in paraformaldehyde for 15 min, followed by staining in Oil Red O (in 70% isopropanol; Shanghai Yuanye Bio-Technology, Shanghai, China) for 40 min and in PBS with Actin-Tracker (50 nM, Beyotime Biotechnology, Shanghai, China) for 30 min, respectively. After that, the cells were washed three times with 70% ethanol and DPBS to remove the excess dye. The nuclei, membrane, and microfilaments of living mature adipose cells that were washed twice with DPBS were stained by PBS with Hoechst 33342 (5 μg ml^−1^, Sigma-Aldrich, Germany), DiO (5 μM, Beyotime Biotechnology, Shanghai, China), and SiR-actin (0.5 μM, Cytoskeleton, USA) for 30 min, respectively (see Fig. S20 of the Supplementary Information). The K562 cells, which were suspended rather than adherent cells, were collected by centrifugation at 1000 r min^−1^ for 5 min and then washed once with PBS to remove the complete medium. The cells were resuspended and the mitochondria were stained by PBS with Mito-Tracker (30 nM, Beyotime Biotechnology, Shanghai, China) for 30 min. After staining, all the staining agents were removed and replaced with phenol red-free complete medium.

The adenoviruses (VectorBuilder, Guangzhou, China) were incubated with amine-reactive Cy5 dyes (1 mg mL^−1^ in DMSO, Shanghai Yuanye Bio-Technology, Shanghai, China) in a carbonate buffer (pH 9.3) at 25 °C for 2 h. The virus mixture was centrifuged at 15000-20000 g for 1-2 h, to remove the unbound Cy5 dyes. The viral aggregates were removed with 0.22 μm pore size filters before experiments. The adipose cells were incubated with the viruses at 37 °C and 5% CO_2_ incubator for 30 min. Cells were washed with DPBS for 2 times before experiments^51,52^.

### Experimental setup

The experimental setup consisted of an inverted fluorescence microscope (Eclipse Ti, Nikon) and a scanning optical tweezing system (OTS) (Tweez 250si, Aresis). The trapping laser (1064 nm) in the OTS was passed through an acousto-optic deflector (AOD) and a beam expander, and was refocused by the inverted objective onto the target. For bright-field imaging, a halogen light source (D-LH/LC:12 V, 100 W) was used to illuminate the sample from the top, and the image was transmitted through the inverted objective (×60, DIC, WD = 2.0, water-immersion, Nikon) and received by a charge-coupled device (CCD) (MG-100, Mshot, Guangzhou, China). For fluorescence imaging, the excitation light from the LED was transmitted through the dichroic mirror and converged by the objective to illuminate the sample. The fluorescent signals were then collected by the objective and received by the CCD and a spectrometer (SHIS VNIR-520-20-S, Wayho Technology, Guangzhou, China). The filtrated bands for exciting the blue (Hoechst 33342), green (DiO, Mito-Tracker and Actin-Tracker), red (FNDs and Oil-red O), and deep red (SiR-actin) markers were 361–389 nm, 465–495 nm, 540–580 nm, and 590–650 nm, respectively.

### Simulations

The electric field distributions of the lipid droplets were simulated with a finite element method (COMSOL Multiphysics, 5.5). The lipid droplets were set as isotropic microspheres. The refractive indices of the lipid droplets and surrounding (cytoplasm) were set as 1.52 and 1.36, respectively. Triangular meshes with a minimum element size of 1.5 nm and a maximum of 100 nm were set for the simulated region (200 μm × 200 μm) with scattering boundary conditions. To calculate the collection efficiency of the lipid droplets, a point source emitter (*λ* = 685 nm) was set in contact with the surface of the lipid droplet. The average Poynting vector flux was computed over a spherical surface (radius: 50 μm) above the droplet^53^, with a center at the emitter. The collection angle was set as ± 47.3° in correspondence to the microscope objective NA (1.0). To numerically analyze the converging ability of the lipid droplet in the non-contact mode, a plane wave (width: 200 μm) at the wavelengths of 473 nm was set as the incident light.

## Supplementary information

Supplementary Information

Three-dimensional (3D) animation of an adipose cell

Optical detection of cancer cells in extracellular fluid

## References

[1] Peng HC (2008). Bioimage informatics: a new area of engineering biology. Bioinformatics.

[2] Kasban H, El-Bendary MAM, Salama DH (2015). A comparative study of medical imaging techniques. Int. J. Inf. Sci. Intell. Syst..

[3] Eggeling C (2018). Advances in bioimaging-challenges and potentials. J. Phys. D: Appl. Phys..

[4] Pierce DW, Vale RD (1998). Single-molecule fluorescence detection of green fluorescence protein and application to single-protein dynamics. Methods Cell Biol..

[5] Derfus AM, Chan WCW, Bhatia SN (2004). Probing the cytotoxicity of semiconductor quantum dots. Nano Lett..

[6] Diaspro, A. et al. *Photobleaching. in Handbook of Biological Confocal Microscopy* 5th edn (ed Pawley, J. B.) Ch. 39, 690–702 (Springer, 2006).

[7] Calabuig A (2017). Investigating fibroblast cells under “safe” and “injurious” blue‐light exposure by holographic microscopy. J. Biophotonics.

[8] Chen LW (2020). Microsphere-toward future of optical microscopes. iScience.

[9] Huang B, Babcock H, Zhuang XW (2010). Breaking the diffraction barrier: super-resolution imaging of cells. Cell.

[10] Zhang X, Liu ZW (2008). Superlenses to overcome the diffraction limit. Nat. Mater..

[11] Wang ZB (2011). Optical virtual imaging at 50 nm lateral resolution with a white-light nanoscope. Nat. Commun..

[12] Chen LW (2018). Remote-mode microsphere nano-imaging: new boundaries for optical microscopes. Opto-Electron. Adv..

[13] Chen XX (2020). Subwavelength imaging and detection using adjustable and movable droplet microlenses. Photonics Res..

[14] Li L (2013). Label-free super-resolution imaging of adenoviruses by submerged microsphere optical nanoscopy. Light.: Sci. Appl..

[15] Wang FF (2016). Scanning superlens microscopy for non-invasive large field-of-view visible light nanoscale imaging. Nat. Commun..

[16] Yang H (2014). Super-resolution biological microscopy using virtual imaging by a microsphere nanoscope. Small.

[17] Yang H, Cornaglia M, Gijs MAM (2015). Photonic nanojet array for fast detection of single nanoparticles in a flow. Nano Lett..

[18] Liang LL (2019). Upconversion amplification through dielectric superlensing modulation. Nat. Commun..

[19] Xing C (2017). Flexible microsphere-embedded film for microsphere-enhanced Raman spectroscopy. ACS Appl. Mater. Interfaces.

[20] Li YC (2016). Manipulation and detection of single nanoparticles and biomolecules by a photonic nanojet. Light.: Sci. Appl..

[21] Miccio L (2021). Optobiology: live cells in optics and photonics. J. Phys.: Photonics.

[22] Franze K (2007). Müller cells are living optical fibers in the vertebrate retina. Proc. Natl Acad. Sci. USA.

[23] Li YC (2017). Enhancing upconversion fluorescence with a natural bio-microlens. ACS Nano.

[24] Li YC, Liu XS, Li BJ (2019). Single-cell biomagnifier for optical nanoscopes and nanotweezers. Light.: Sci. Appl..

[25] Miccio L (2015). Red blood cell as an adaptive optofluidic microlens. Nat. Commun..

[26] Wu TL (2020). Waveguiding and focusing in a bio-medium with an optofluidic cell chain. Acta Biomaterialia.

[27] Humar M, Yun SH (2015). Intracellular microlasers. Nat. Photonics.

[28] Stavenga DG, Wilts BD (2014). Oil droplets of bird eyes: microlenses acting as spectral filters. Philos. Trans. R. Soc. B: Biol. Sci..

[29] Cho SY (2021). Cellular lensing and near infrared fluorescent nanosensor arrays to enable chemical efflux cytometry. Nat. Commun..

[30] Miccio L (2019). Biological lenses as a photomask for writing laser spots into ferroelectric crystals. ACS Appl. Bio Mater..

[31] Monks JN (2016). Spider silk: mother nature’s bio-superlens. Nano Lett..

[32] Farese RV, Walther TC (2009). Lipid droplets finally get a little R-E-S-P-E-C-T. Cell.

[33] Okumura T (2011). Role of lipid droplet proteins in liver steatosis. J. Physiol. Biochem..

[34] Soppina V (2009). Tug-of-war between dissimilar teams of microtubule motors regulates transport and fission of endosomes. Proc. Natl Acad. Sci. USA.

[35] Thiam AR, Beller M (2017). The why, when and how of lipid droplet diversity. J. Cell Sci..

[36] Blázquez-Castro A (2019). Optical tweezers: phototoxicity and thermal stress in cells and biomolecules. Micromachines.

[37] Luby-Phelps K (1999). Cytoarchitecture and physical properties of cytoplasm: volume, viscosity, diffusion, intracellular surface area. Int. Rev. Cytol..

[38] Hao X (2011). Microsphere based microscope with optical super-resolution capability. Appl. Phys. Lett..

[39] Wang, Z. B. in *Nanoscience* Vol. 3 (eds O’Brien, P. & Thomas, P. J.) 193–210 (The Royal Society of Chemistry, 2016).

[40] Yang H (2016). Super-resolution imaging of a dielectric microsphere is governed by the waist of its photonic nanojet. Nano Lett..

[41] Yu SJ (2005). Bright fluorescent nanodiamonds: no photobleaching and low cytotoxicity. J. Am. Chem. Soc..

[42] Li XH (2019). Enhancement of the second harmonic generation from WS_2_ monolayers by cooperating with dielectric microspheres. Adv. Optical Mater..

[43] Stelzer EHK (1998). Contrast, resolution, pixelation, dynamic range and signal-to-noise ratio: fundamental limits to resolution in fluorescence light microscopy. J. Microsc..

[44] Takaoka M (2010). Endovascular injury induces rapid phenotypic changes in perivascular adipose tissue. Arteriosclerosis Thrombosis Vasc. Biol..

[45] Jodalen H, Lie R, Rotevatn S (1982). Effect of isoproterenol on lipid accumulation in myocardial cells. Res. Exp. Med..

[46] den Hartigh LJ (2010). Fatty acids from very low-density lipoprotein lipolysis products induce lipid droplet accumulation in human monocytes. J. Immunol..

[47] Lee LL (2017). Triglyceride-rich lipoprotein lipolysis products increase blood-brain barrier transfer coefficient and induce astrocyte lipid droplets and cell stress. Am. J. Physiol.-Cell Physiol..

[48] Kim SW (2020). Eicosapentaenoic acid (EPA) activates PPARγ signaling leading to cell cycle exit, lipid accumulation, and autophagy in human meibomian gland epithelial cells (hMGEC). Ocul. Surf..

[49] Ives JT, Normann RA, Barber PW (1983). Light intensification by cone oil droplets: electromagnetic considerations. J. Optical Soc. Am..

[50] Zhou S (2017). Effects of whispering gallery mode in microsphere super-resolution imaging. Appl. Phys. B.

[51] Lakadamyali M (2003). Visualizing infection of individual influenza viruses. Proc. Natl Acad. Sci. USA.

[52] Xu HJ (2015). Real-time imaging of rabies virus entry into living vero cells. Sci. Rep..

[53] Gérard D (2009). Efficient excitation and collection of single-molecule fluorescence close to a dielectric microsphere. J. Optical Soc. Am. B.

