# Supplementary material for: Lipid droplets as endogenous intracellular microlenses
Source: Light Sci Appl. 2021 Dec 6;10:242. doi: 10.1038/s41377-021-00687-3 (PMC8648767; doi:10.1038/s41377-021-00687-3)
Supplement: Supplementary file 1 — Supplementary Information [file 41377_2021_687_MOESM1_ESM.docx]

**Supplementary Information for**

**Lipid droplets as endogenous intracellular microlenses**

Xixi Chen^1^, Tianli Wu^1^, Zhiyong Gong^1^, Jinghui Guo^2^, Xiaoshuai Liu^1^, Yao Zhang^1,^*, Yuchao Li^1,^*, Pietro Ferraro^3,^*, and Baojun Li^1,^*

^1^Institute of Nanophotonics, Jinan University, Guangzhou 511443, China

^2^Department of Physiology, School of Medicine, Jinan University, Guangzhou 510632, China

^3^CNR-ISASI, Institute of Applied Sciences and Intelligent Systems «E. Caianiello», Via Campi Flegrei 34, 80078 Pozzuoli, Naples

*Corresponding authors. Email: [zhyao5@jnu.edu.cn](mailto:zhyao5@jnu.edu.cn) (Y.Z.), [liyuchao@jnu.edu.cn](mailto:liyuchao@jnu.edu.cn) (Y.L.), [pietro.ferraro@cnr.it](mailto:pietro.ferraro@cnr.it) (P.F.), or [baojunli@jnu.edu.cn](mailto:baojunli@jnu.edu.cn) (B.L.)

**S1 Materials and Methods**

***S1.1 Measurement of refractive indices***

The refractive indices *n* of the lipid droplets and the cytoplasm from adipocytes was measured by an index matching method using an optical microscope in differential interference contrast (DIC) mode^S1-S3^. The culture medium of the mature adipocytes (12 days after differentiation) was replaced with refractive index matching (RIM) liquid that was mixed with undiluted liquid in two different indices (*i.e.* *n*_1_ = 1.48 and *n*_2_ = 1.64) (Cargille Laboratories, USA) and ~0.1% Triton X-100 (Sigma, USA) for permeabilizing the cell membrane to release the lipid droplets. The droplets were immersed by the RIM liquid the index of which was gradually increased, and the index of the lipid droplets was then measured using an Abbe-type refractometer (Shenguang, Shanghai, China). The droplets became invisible in the DIC image at *n* = 1.52 ± 0.03 of the RIM liquid which was taken as the index of the droplets. Similarly, the index of the cytoplasm was measured by injecting a glycerol solution (99.9%, Reagent grade, Beyotime Biotechnology, Shanghai, China) with varying indices into cells under DIC imaging. The index of the cytoplasm was matched when the glycerol solution was almost invisible at *n* = 1.36 ± 0.01.

***S1.2 Preparation of the fluorescent nanodiamonds (FNDs)***

Commercially available water-soluble FNDs (5.5 × 10^4^ particles μl^-1^, ~300 NV centers per particle, excitation wavelength: 570 nm) (Adamas Nanotechnologies, USA) were used as a sample for signal enhancement. Deionized water (2 ml) was added to the FNDs solution (5 μl) at 24 °C, and the solution was shaken with the ultrasound at a frequency of 50 kHz for 15 mins. The mixed solution was dropped onto the surface of a glass slide and evaporated for 3 hours to form the FNDs for the signal enhancement experiments *in vitro*.

***S1.3*** ***Simulation of transversely polarized dipole***

The transversely polarized dipole was set as a point source (emitter) in a medium with a refractive index of 1.36. The lipid droplets were set as isotropic microspheres with a refractive index of 1.52 (supplementary Fig. S6). The collection efficiencies were calculated as 42.1% and 48.0% for the emitters without and with the lipid droplet (*D* = 9 μm) (Fig. S6a, b), respectively. The enhancement factor *f*_1_ for the collection efficiency, defined as the ratio of the collection efficiencies with the droplets (*D* from 1 to 20 μm) to those without the droplets, was estimated and presented by the purple dots in Fig. S6c. In this case, as the direction of dipole was parallel to the direction of the objective, most of the light has been collected directly by the objective and the improvement in collection efficiency by the lipid droplets is much weaker than that in the longitudinally polarized dole (Fig. 2f).

***S1.4 Velocity of the moving lipid droplets***

In the solution, the freely suspended lipid droplets trapped by the optical trap can move in synchrony with the motion of the trap, as long as the trapping force (*F*_T_) on the lipid droplet was not exceeded by a drag force (*F*_S_) from the flow. According to Stokes’ law, *F*_S_ can be expressed as *F*_S_ = 3πη*vD*, where η is the viscosity coefficient of water (0.9 × 10^-4^ Pa·s), *v* is the velocity of the sphere relative to the water, and *D* is the diameter of the sphere. When moved to the target position, the velocity *v* of the trapped lipid droplet depends on the power of the trapping laser, the diameter of the lipid droplet and the speed of the trap. The velocity of the trapped lipid droplet is then considered as the maximum speed (*v*_max_) of the trap when the displacement (Δ*d*) from the center of the optical trap to the center of the moving lipid droplet is equal to the radius of the droplet. According to the formula *F*_T_ = K·Δ*d* = *F*_S_ = 3πη*vD*, where K is trap stiffnesses of the lipid droplets, the values of *v*_max_ were calculated, as shown in supplementary Fig. S12. However, due to the much stronger drag force caused by the crowded intracellular environment, the movement of the trapped lipid droplets to the target position inside the cells becomes much slower (0.001 to 5 μm s^-1^).

***S1.5 Comparison on the lensing effects between lipid droplets and*** ***other bio-microlenses***

The refractive index of the lipid droplets in this work (*n* = 1.49-1.55) is higher than those of chloroplasts (*n* = 1.42)^S4^ and RBCs (*n* = 1.38-1.42)^S5^, which implies a better imaging and light converging ability because the microlenses with a higher index and a smaller radius of curvature generally hold stronger lensing effect. In our experiments, the structures of the gratings (pitch: 1 μm, width: 650 nm and 350 nm) and the Blu-ray Disk (BD) (pitch: 300 nm, width: 100 nm and 200 nm) were imaged and the signals from fluorescent nanodiamonds were enhanced by a chloroplast, an RBC, and a lipid droplet (Fig. S15). It can be seen that on the same condition, the lipid droplet with a higher index and a nearly spherical shape provided a better performance in magnification and fluorescence enhancement, which enables the resolving of 100 nm feature size.

**S2 Supplementary Figures**

**
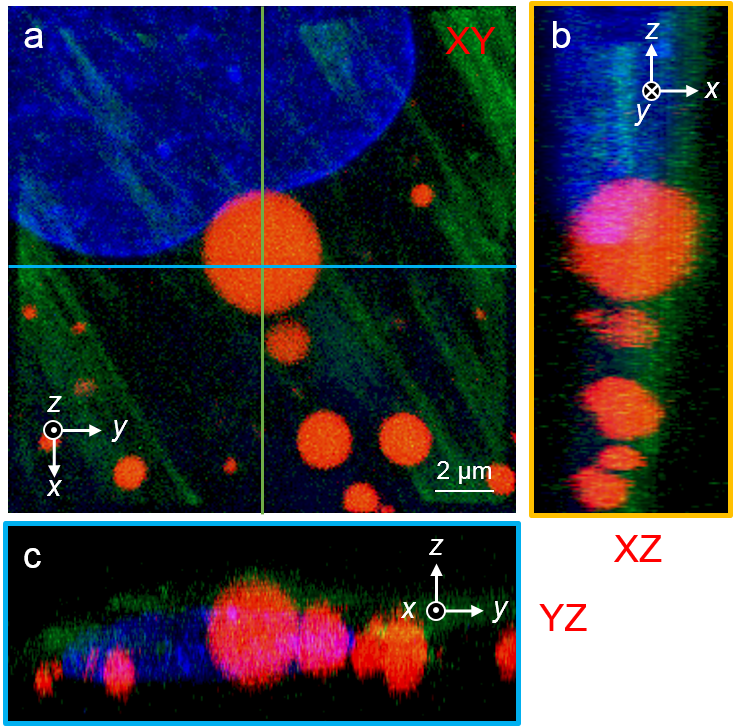
**

**Fig. S1.** **3D reconstruction (Z-stack) from a fixed adipose cell**. The nucleus, lipid droplets, and microfilaments were indicated by Hoechst 33342 (blue), Oil-red O (red), and Actin-Tracker (green) using a confocal fluorescence microscope (63× magnification, LSM 800, Zeiss). The XY (**a**), XZ (**b**) and YZ (**c**) projections show the spherical shape of the lipid droplets.

**
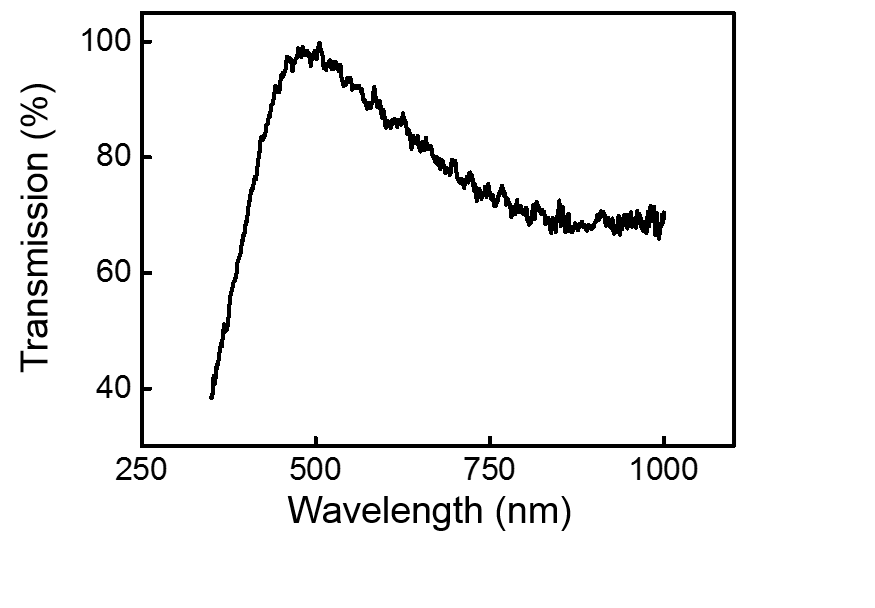
**

**Fig. S2. Transmittance spectrum of the lipid droplets.**


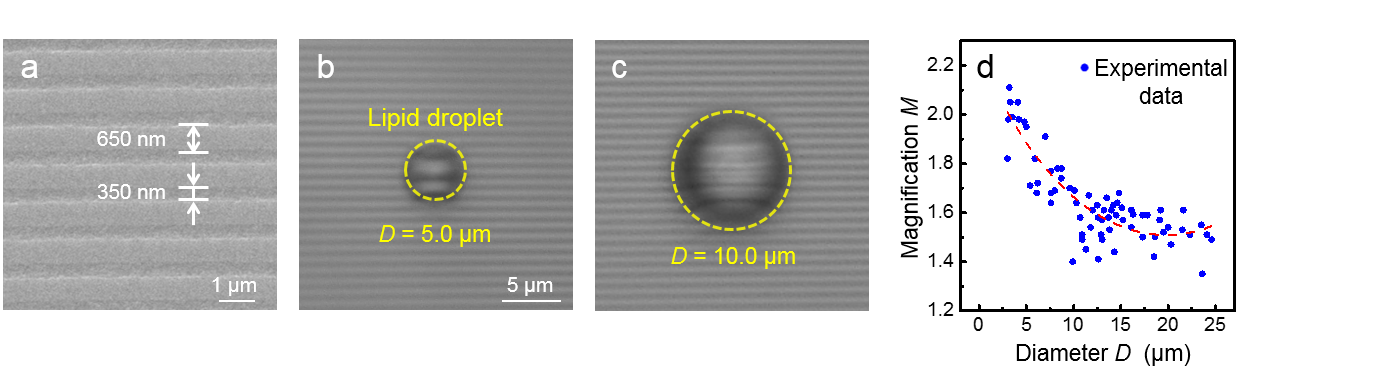


**Fig. S3. Imaging of gratings with the lipid droplets** **in the RIM liquid (*n* = 1.36).** (**a**) SEM image of the gratings (pitch: 1 μm, width: 650 nm and 350 nm). (**b**, **c**) Optical microscope images of the gratings with the assistance of the lipid droplets with diameters of (**b**) 5.0 and (**c**) 10.0 μm. (**d**) Magnification *M* of the lipid droplets as a function of diameter *D*.


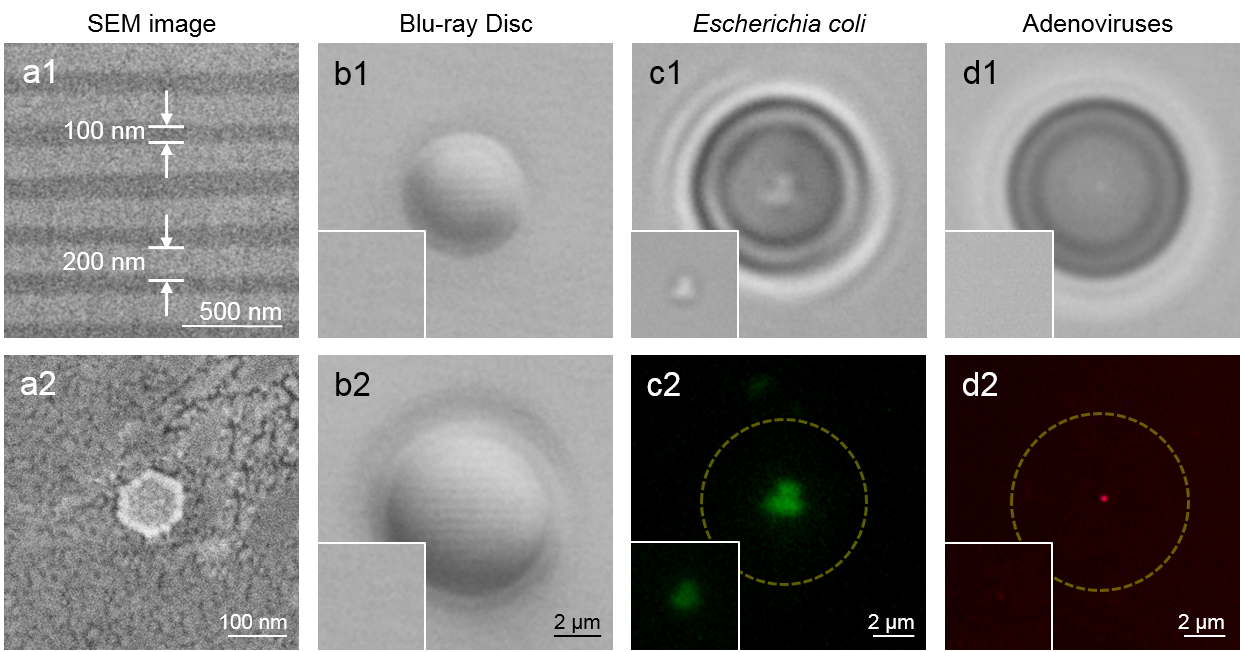


**Fig. S4. Imaging of grating structures of a Blu-ray Disk (BD), *Escherichia coli* (*E. coli*), and adenoviruses *in vitro* in the RIM liquid (*n* = 1.36). (a)** SEM images of the gratings of a BD (**a1**) and adenovirus (**a2**). (**b**) Optical microscope images of the BD gratings with the presence of the lipid droplets with diameters of 4.6 (**b1**) and 9.7 (**b2**) μm. The insets show the images of the BD without the lipid droplets. (**c**) Optical microscope images of the GFP-*E. coli* with the presence of the lipid droplet with a diameter of 8.0 μm in bright field (**c1**) and fluorescence (**c2**). The insets show the images of the GFP-*E. coli* without the lipid droplets. (**d**) Optical microscope images of the Cy5-labeled adenoviruses with the presence of the lipid droplet with a diameter of 8.5 μm in bright field (**d1**) and fluorescence (**d2**). The insets show the images of the adenoviruses without the lipid droplets.

**
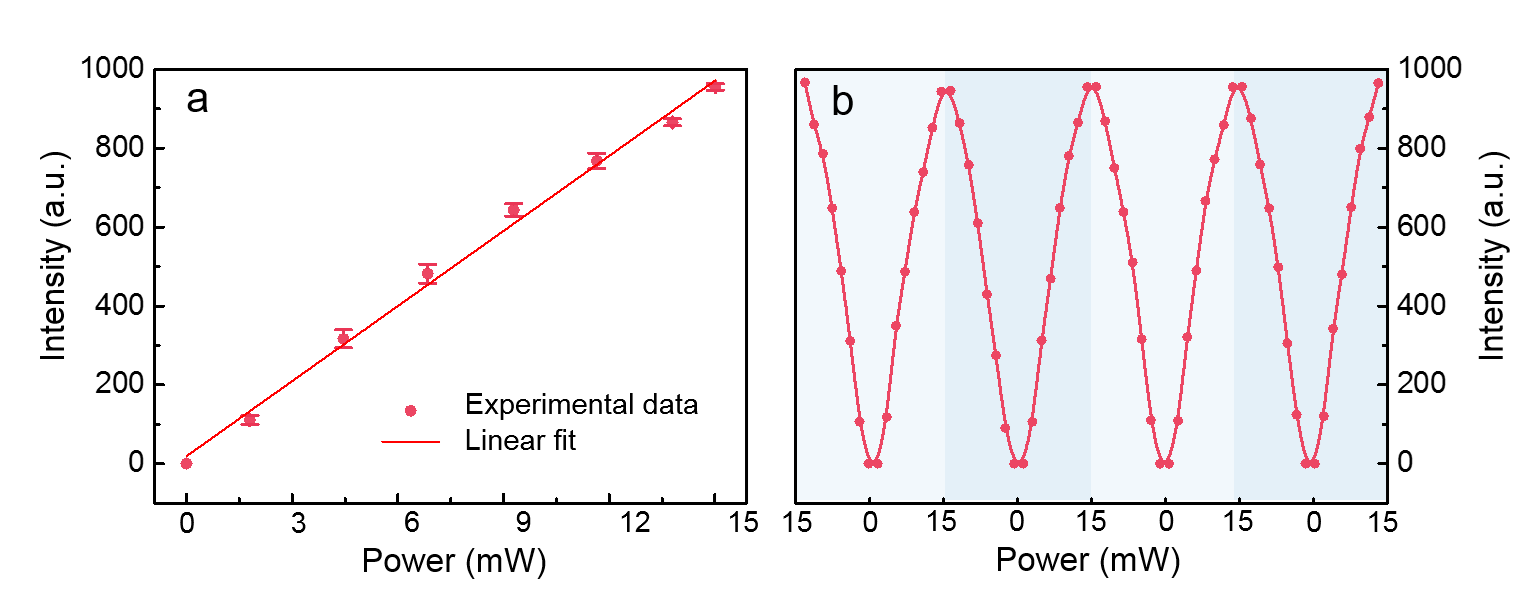
**

**Fig. S5.** **Fluorescent properties of the FNDs.** (**a**) Fluorescence intensity of the FNDs as a function of the power of excitation light (540-580 nm). (**b**) Photostability of the FNDs obtained by repeating the excitation of the fluorescence for four times.

**
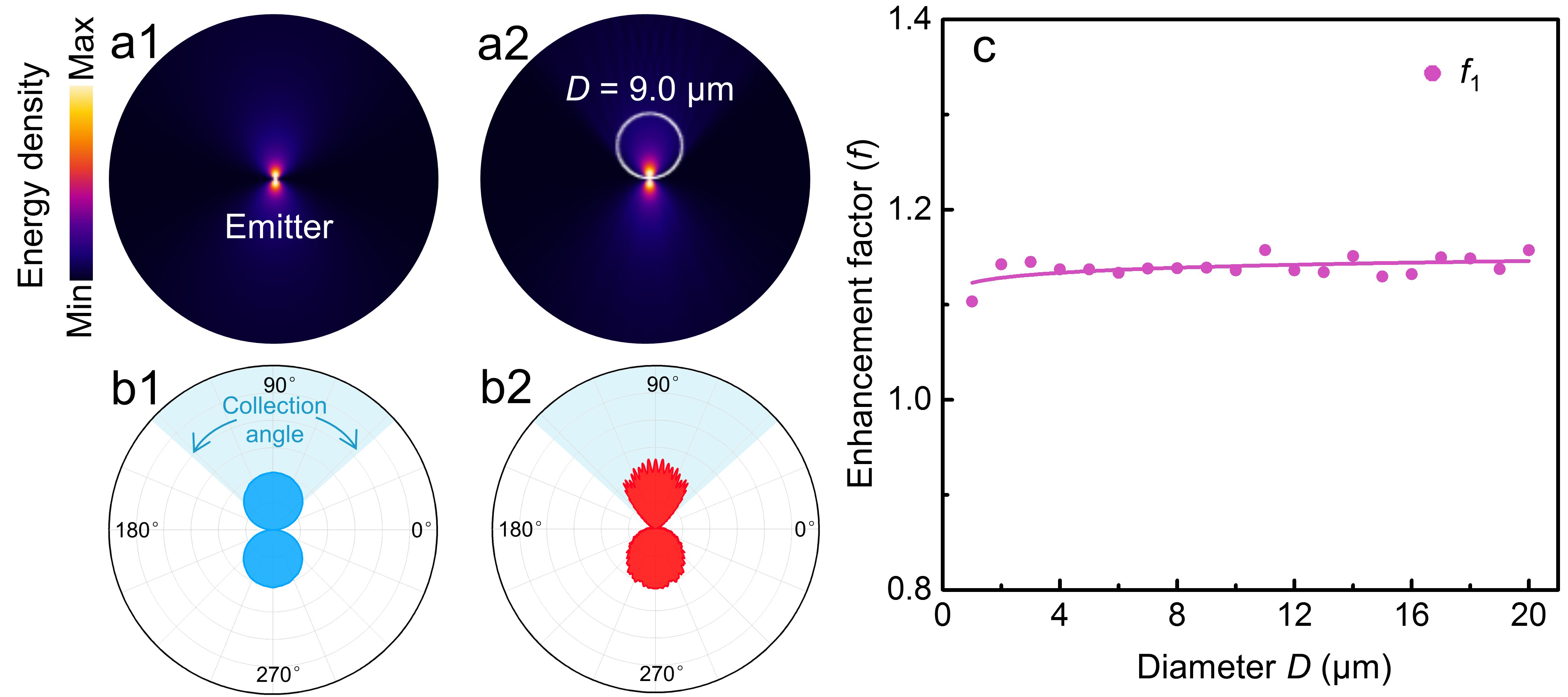
**

**Fig. S6.** **Simulation of transversely polarized dipole.** (**a**) Energy density distributions without (**a1**) and with (**a2**) the lipid droplet (*D* = 9 μm). (**b**) The corresponding emission patterns. (**c**) Enhancement factor *f*_1_ for the collection efficiency as a function of the diameter of the lipid droplets.


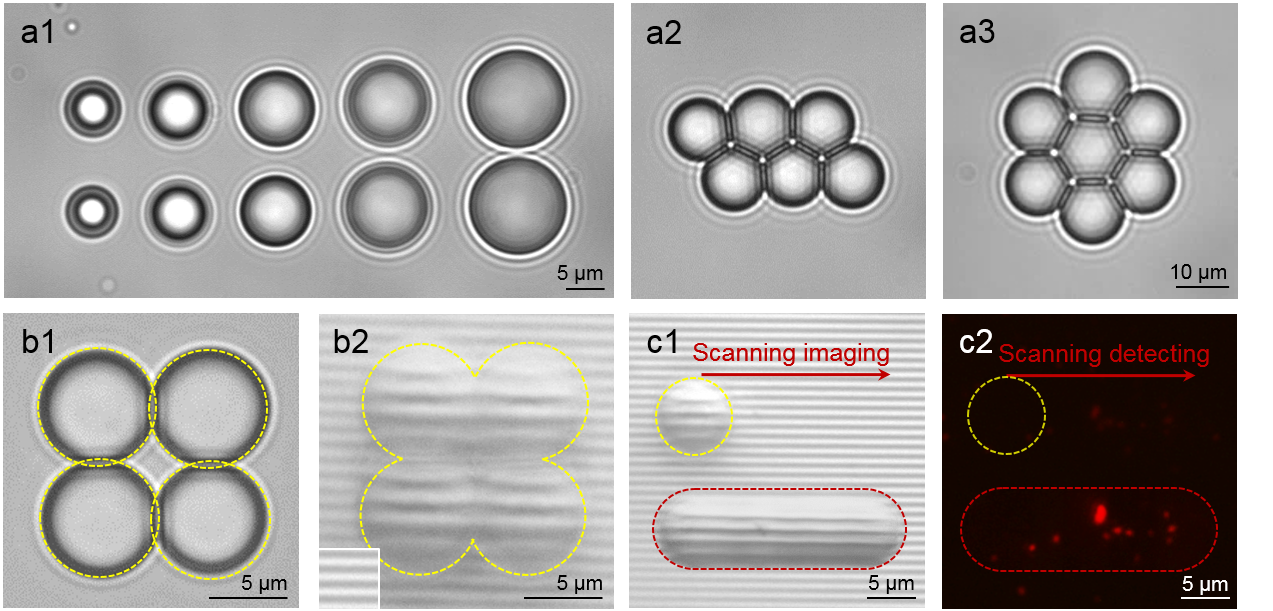


**Fig. S7.** **2D microlens arrays of lipid droplets and scanning imaging with a moving lipidic microlens.** (**a**) Optical microscope images of a 5 × 2 array of lipid droplets with diameters of 7.7-12.3 μm arranged in ascending order (**a1**), a 3 × 2 array of lipid droplets with the same diameter (7.0 ± 0.3 μm) (**a2**), and a hexagonal close-packed array of the lipid droplets with the same diameter (7.0 ± 0.3 μm) (**a3**). (**b**) Imaging of gratings (pitch: 1 μm, width: 650 nm and 350 nm) by a 2 × 2 array of lipidic microlenses with the same diameter (7.0 ± 0.3 μm) in the RIM liquid (*n* = 1.36). (**c**) Scanning imaging of the gratings (**c1**) and scanning detecting of the fluorescent nanodiamonds (**c2**) obtained by moving a single lipid droplet. The red dotted outline shows the image reconstructed by stitching the signals from the virtual image plane of the lipid droplet.

**
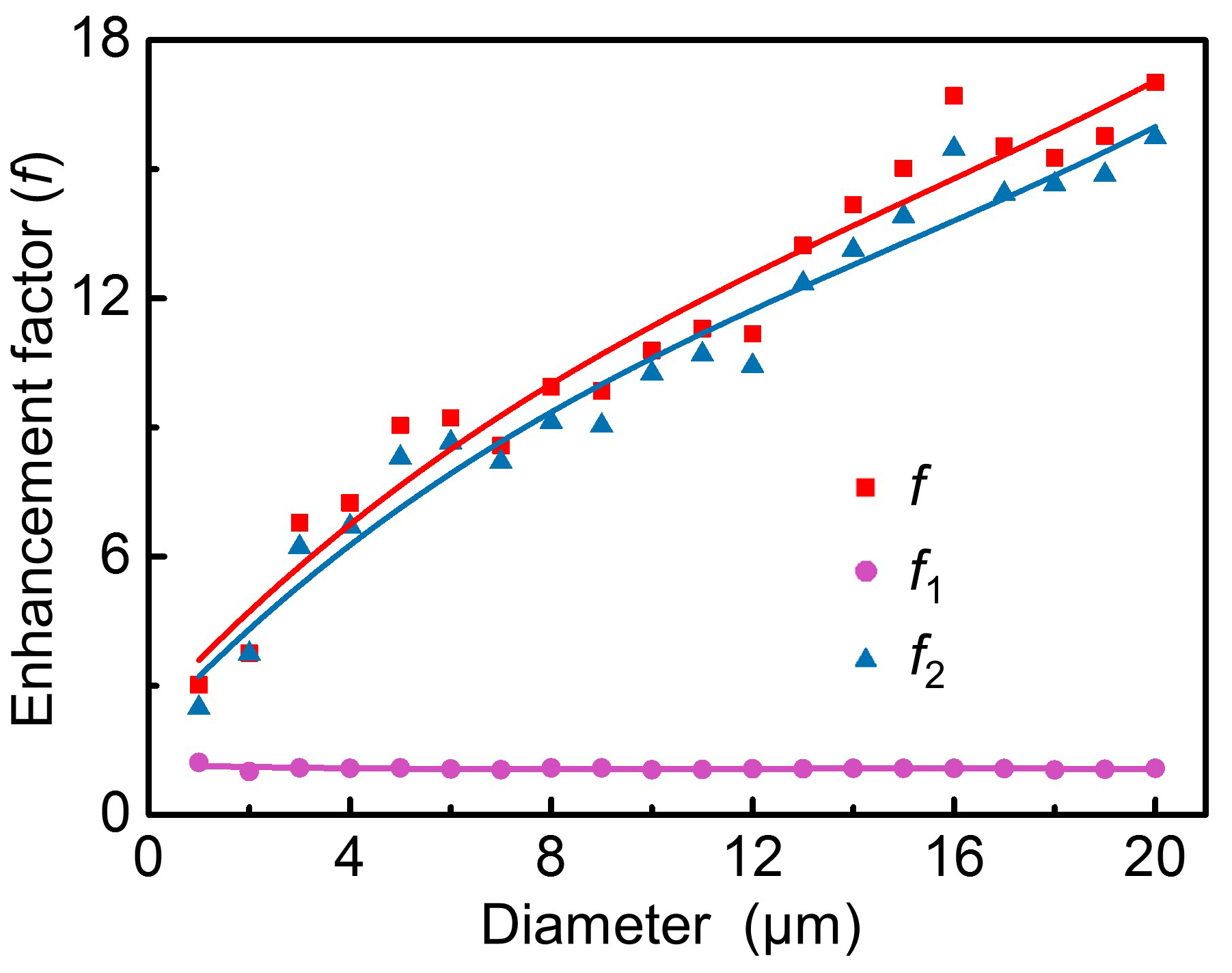
**

**Fig. S8.** **Enhancement factor (*f*) in the focus as a function of the diameters *D* of lipid droplets.** *f* is the product of *f*_1_ and *f*_2_ where *f*_1_ is the factor for the collection efficiency while *f*_2_ is that for intensity enhancement of the excitation light.

**
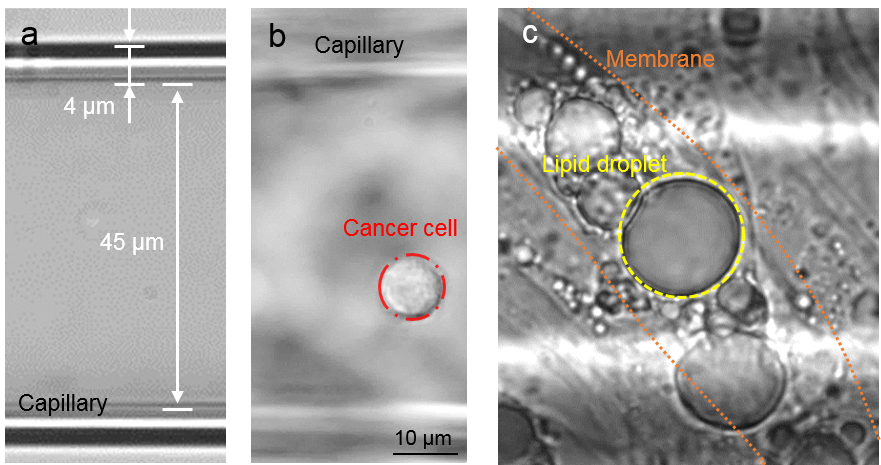
**

**Fig. S9. Optical microscope images of the cells in the capillary.** (**a**) The axial cross-sectional view of the capillary (inner diameter: 45 μm, wall thickening: 4 μm). (**b**) The suspended cancer cell (K562) in the capillary. (**c**) The adipose cell attached to capillary with a lipid droplet (*D* = 20.0 μm) inside the cell.

**
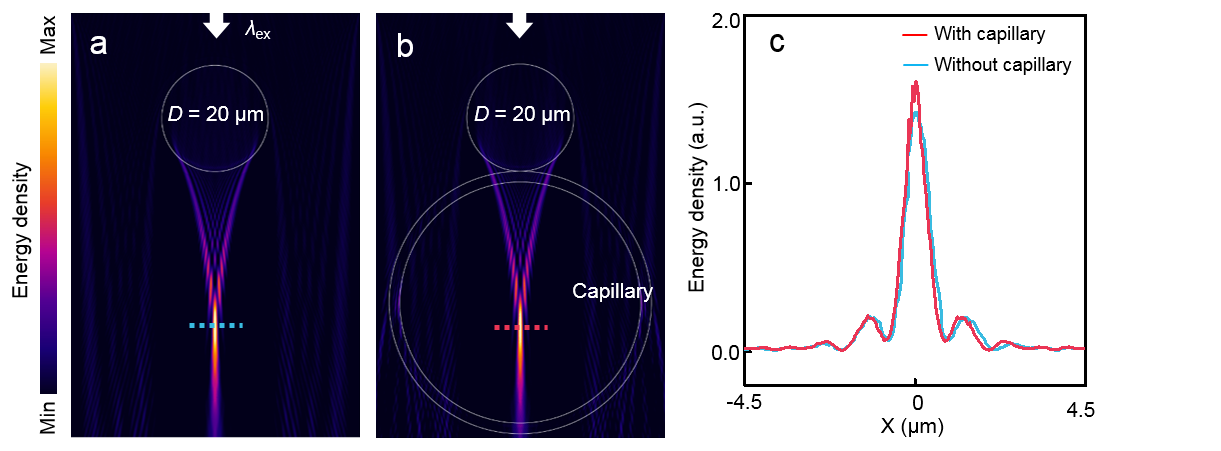
**

**Fig. S10. Energy density distributions for the lipid droplets.** (**a**) Energy density of a lipid droplet (*D* = 20.0 μm) at the excitation wavelength of 475 nm in free space. (**b**) Energy density of the droplet near the capillary. (**c**) Energy density distributions along the observation lines through the focus in (**a**) (blue) and (**b**) (red).

**
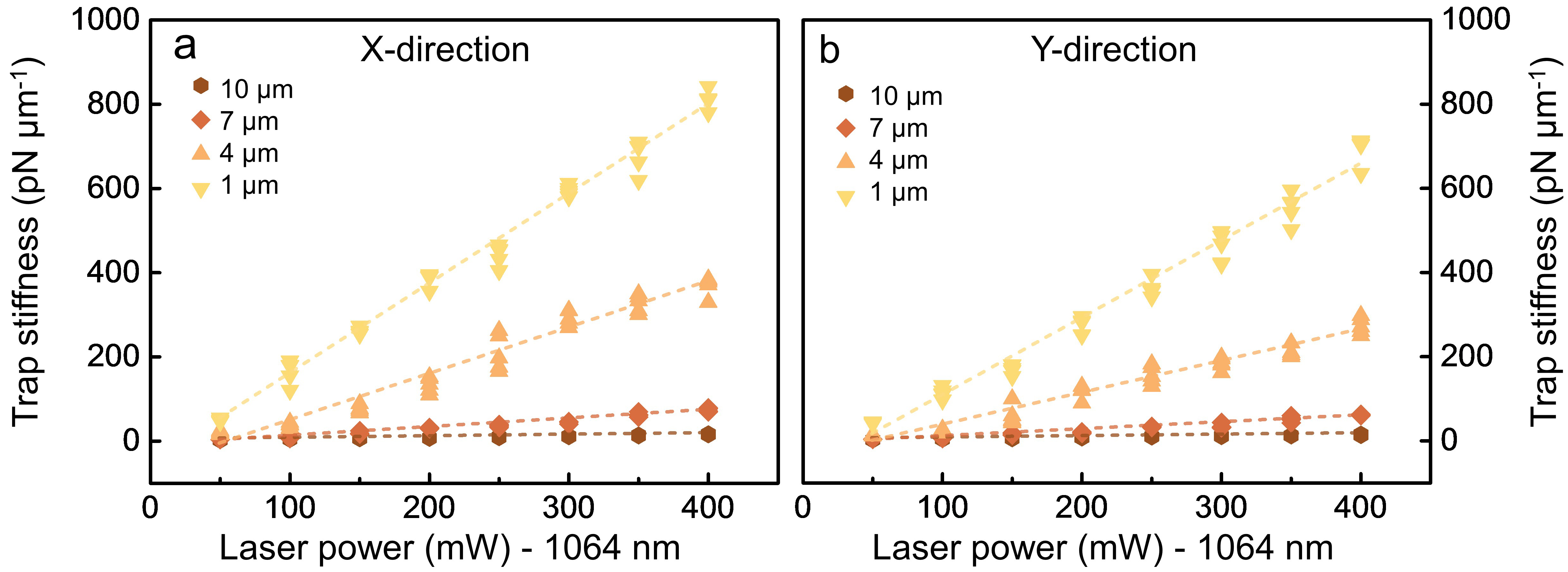
**

**Fig. S11.** **Trap stiffnesses of the lipid droplets.** (**a**) Stiffness as a function of the laser power along X-direction of the trap in the RIM liquid (glycerol solution, *n* = 1.36). (**b**) Stiffness as a function of the laser power along Y-direction.

**
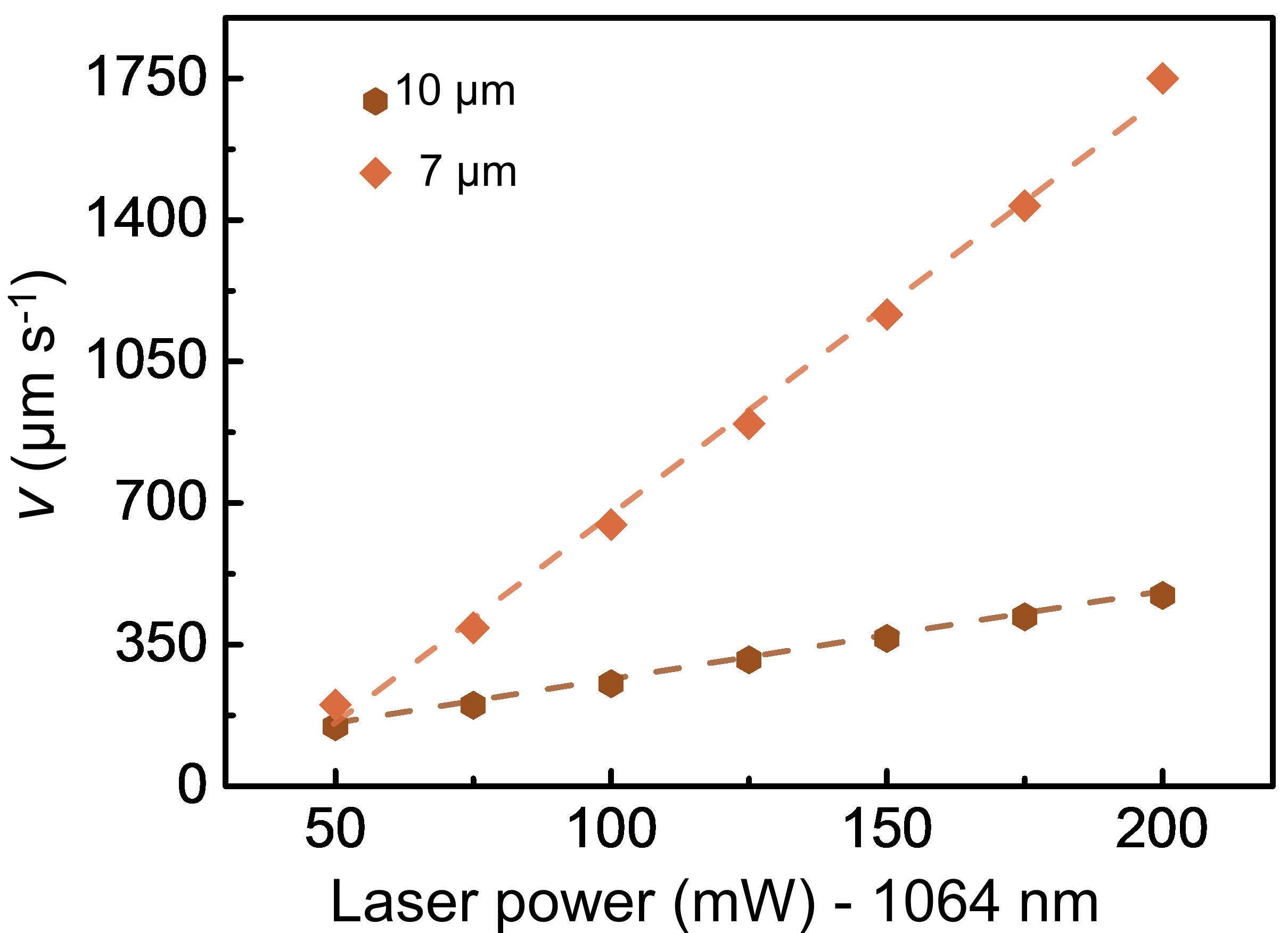
**

**Fig. S12. Calculated maximum velocity (*v*) of the lipid droplets with diameters of 7 (orange diamonds) and 10 μm (brown hexagons) as functions of the laser power.**

**
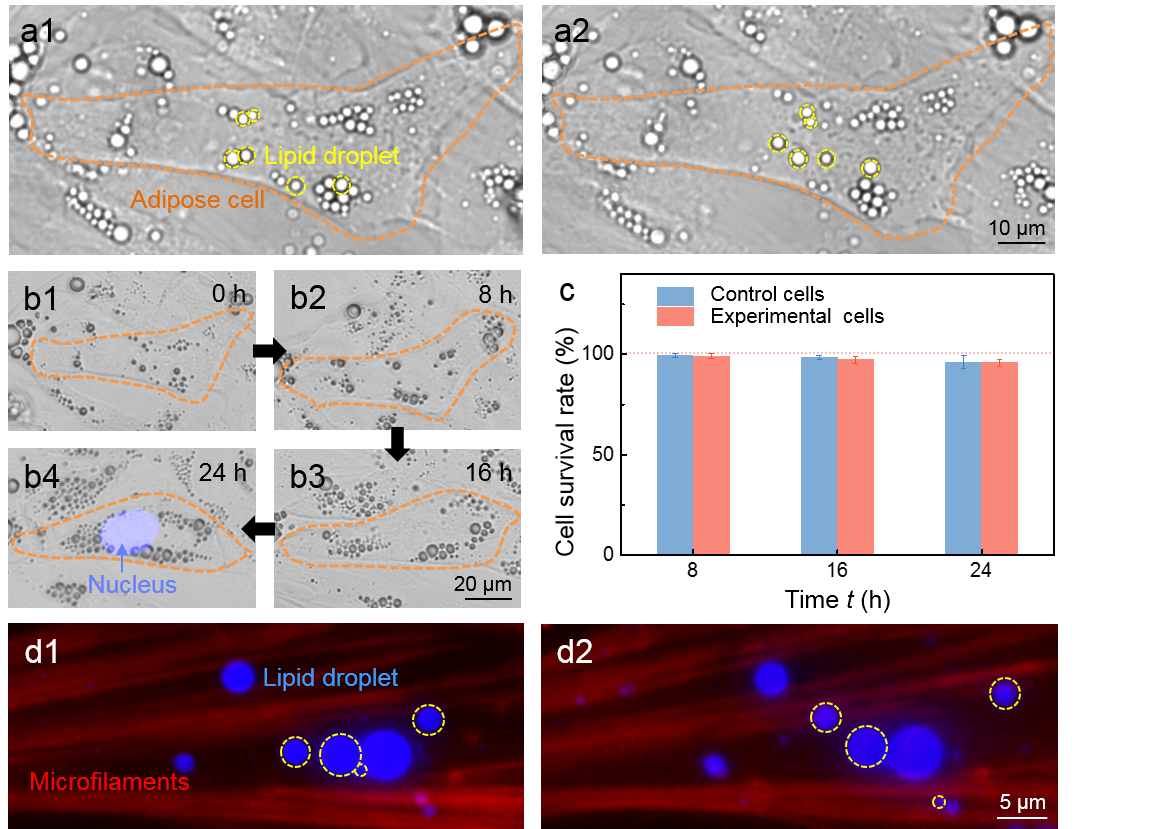
**

**Fig. S13.** **Cell viability after trapping and moving of the lipid droplets in adipose cells.** (**a**) Optical microscope images of adipose cell before (**a1**) and after (**a2**) moving the lipid droplets. The yellow dashed circles represent the moved lipid droplets. (**b**) Images recorded every 8 h. (**c**) Cell survival rate at 24 h in untreated cells (control cells) and treated cells (experimental cells). (**d**) Fluorescence images of an adipose cell before (**d1**) and after (**d2**) moving the lipid droplets. The lipid droplets were labeled blue and the microfilaments were labeled red.

**
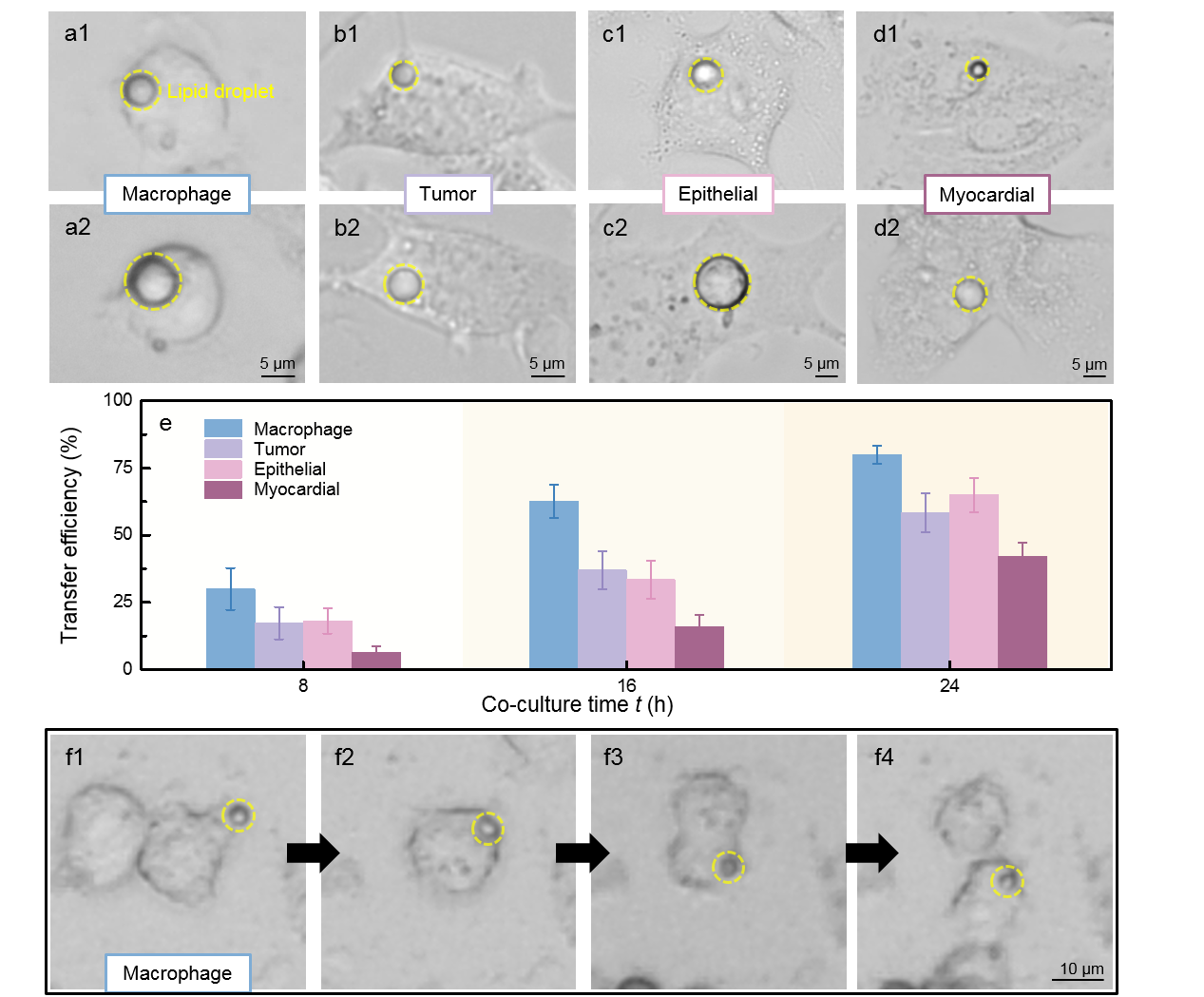
**

**Fig. S14. Co-culture of the lipid droplets with other cells for 8-24 hours.** (**a-d**) Optical microscope images of co-culture of the lipid droplets with (**a**) macrophage, (**b**) tumor, (**c**) epithelial, and (**d**) myocardial cells for 24 hours. (**e**) Histogram of transfer efficiency in the internalization of the lipid droplets by the four types of cells. (**f**) The macrophage cell exhibited a proliferation after the internalization of the lipid droplet.


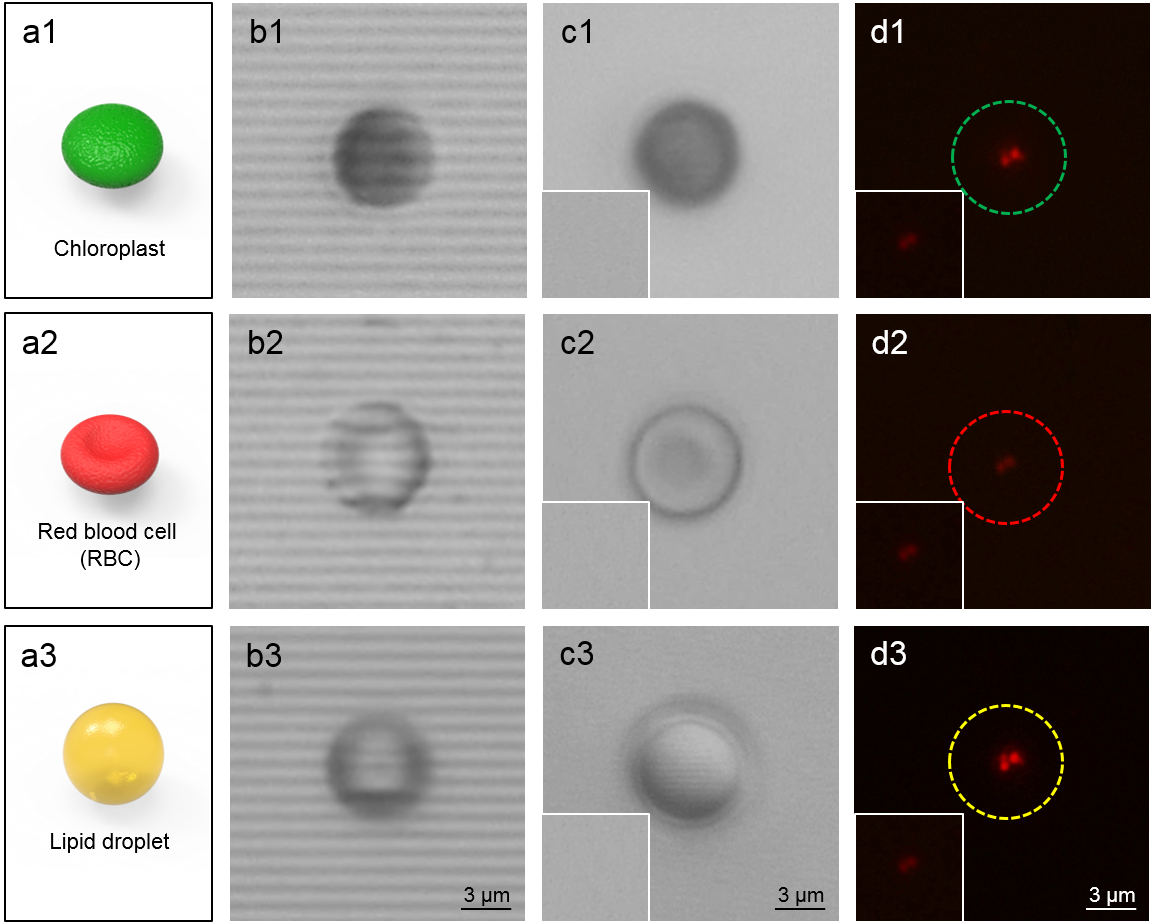


**Fig. S15.** **Comparison on the lensing effects between lipid droplets and other bio- microlenses.** (**a**) Schematics of chloroplast (**a1**), RBC (**a2**) and lipid droplet (**a3**). (**b**) Optical microscope images of the gratings (pitch: 1 μm, width: 650 nm and 350 nm) by the assistance of a 6.0-μm-diameter chloroplast (**b1**), a 6.8-μm-diameter RBC (**b2**) and a 6.2-μm-diameter lipid droplet (**b3**). (**c**) Optical microscope images of a Blu-ray disk (BD) by the assistance of a 6.0-μm-diameter chloroplast (**c1**), a 6.6-μm-diameter RBC (**c2**) and a 6.3-μm-diameter lipid droplet (**c3**). The insets show the images of the BD. (**d**) Fluorescence images of fluorescent nanodiamonds by the chloroplast (**d1**), RBC (**d2**) and lipid droplet (**d3**). The the insets show the images of the FNDs without the presence of the microlenses.

**
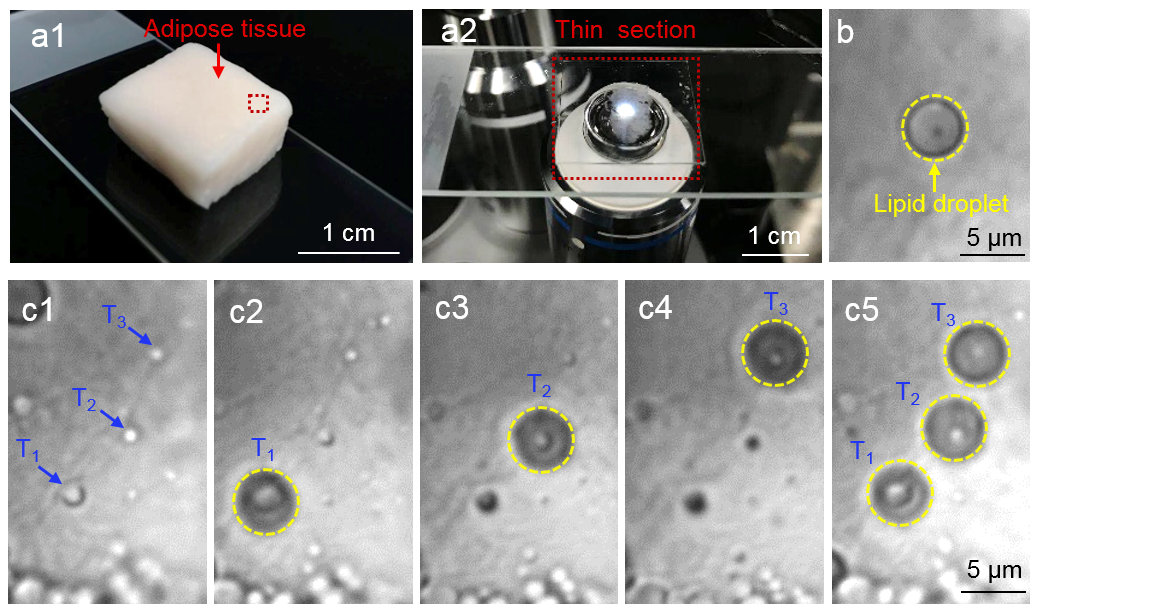
**

**Fig. S16. Lipid droplets on porcine adipose tissues.** (**a**) The bulky adipose tissue (**a1**) and the tissue sections (**a2**) used in the experiments. (**b**) A lipid droplet (5 μm) free from the adipose tissue was trapped by the optical tweezers. (**c**) The lipid droplet was moved for imaging the targets T_1_, T_2_, and T_3_) on the tissue surface. A simultaneous imaging of the three targets was also achieved by trapping and moving three lipid droplets.


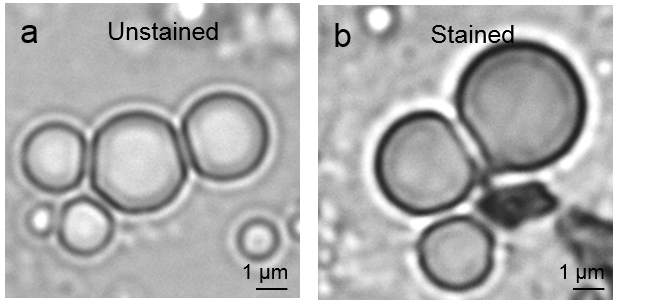


**Fig. S17. Optical microscope images of the lipid droplets unstained** (**a**) **and stained** (**b**) **with lipid-soluble pigments in living cells.** The refractive index of these lipid droplets can be increase by up to 1.6 by binding lipid-soluble pigments.


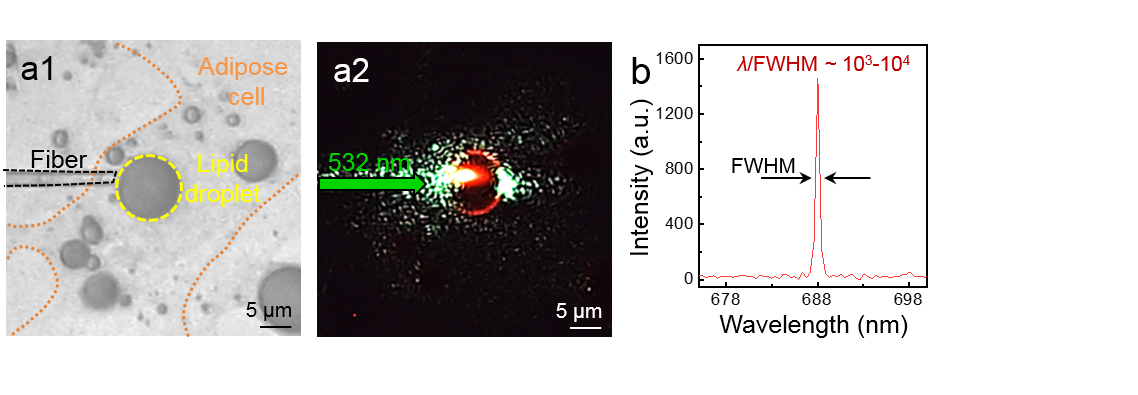


**Fig. S18. Lipid droplets acting as microcavities to support resonances in whispering gallery modes (WGMs). (a)** Images of a stained the lipid droplet (diameter: 15.1 μm) inside an adipose cell with an optical fiber probe next to it in bright field (**a1**) and fluorescence (**a2**). (**b**) Spectrum of the fluorescence signals showing a WGM resonance from the lipid droplet.

**
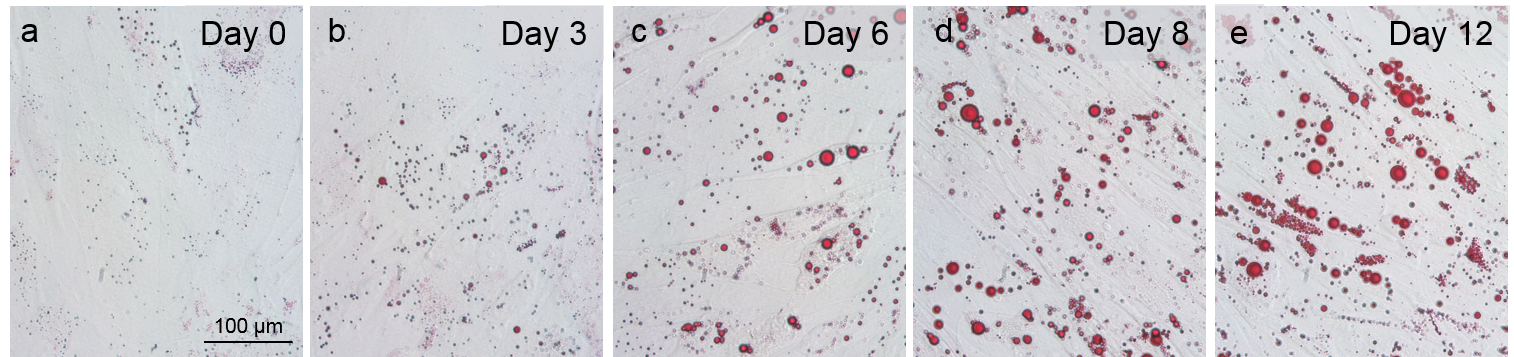
**

**Fig. S19. Adipocyte differentiation and lipid droplets accumulation.** (**a**) Pre-adipocytes grew to confluence, and were then induced to differentiate in the defined medium for 12 days until maturing into adipocytes. The lipid droplets were labeled in Oil-red O (red) in the adipocytes on days 0 (**a**) 3 (**b**), 6 (**c**), 8 (**d**), and 12 (**e**), indicating the differentiation induction.

**
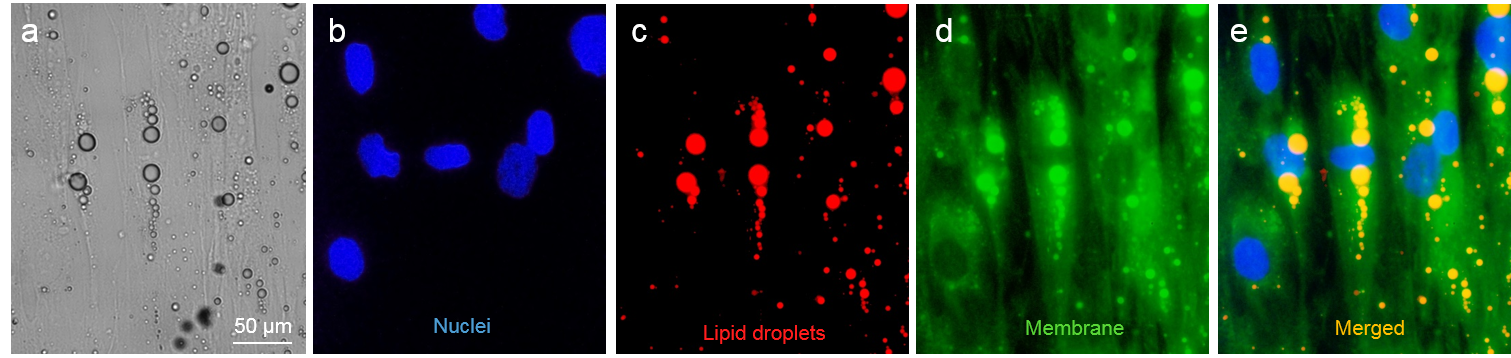
**

**Fig. S20. Bright-field and fluorescence imaging of the adipose cells.** (**a**) Bright-field images of the fixed adipose cells. (**b**) Nuclei indicated by Hoechst 33342 (blue). (**c**) Lipid droplets indicated by Oil-red O (red). (**d**) Membrane indicated by DiO (green). (**e**) Merged image of **b**-**d**.

**References**

S1 Soppina, V. *et al*. Tug-of-war between dissimilar teams of microtubule motors regulates transport and fission of endosomes. *Proceedings of the National Academy of Sciences of the United States of America* **106**, 19381-19386 (2009).

S2 Leidel, C. *et al*. Measuring molecular motor forces *in vivo*: implications for tug-of-war models of bidirectional transport. *Biophysical Journal* **103**, 492-500 (2012).

S3 Ives, J. T., Normann, R. A. & Barber, P. W. Light intensification by cone oil droplets: electromagnetic considerations. *Journal of the Optical Society of America* **73**, 1725-1731 (1983).

S4 Berdnik, V. V. & Mukhamedyarov, R. D. Radiative transfer in plant leaves. *Optics and* *Spectrosco*py **90**, 580-591 (2001).

S5 Miccio, L. *et al*. Red blood cell as an adaptive optofluidic microlens. *Nature Communications* **6**, 6502 (2015).
